# Building a human pluripotent stem cell-based gonadal niche: improving *in vitro* systems with *in vivo* insights

**DOI:** 10.1093/humupd/dmaf012

**Published:** 2025-06-28

**Authors:** Mathangi Lakshmipathi, Nina Dartée, Arina Puchkina, Madalena Vaz Santos, Ilse J de Bruin, Geert Hamer, Ans M M van Pelt, Susana M Chuva de Sousa Lopes, Callista L Mulder, Willy M Baarends

**Affiliations:** Reproductive Biology Laboratory, Center for Reproductive Medicine, Amsterdam UMC, University of Amsterdam, Amsterdam, The Netherlands; Amsterdam Reproduction and Development Research Institute, Amsterdam, The Netherlands; Department of Developmental Biology, Erasmus MC, Rotterdam, The Netherlands; Department of Developmental Biology, Erasmus MC, Rotterdam, The Netherlands; Reproductive Biology Laboratory, Center for Reproductive Medicine, Amsterdam UMC, University of Amsterdam, Amsterdam, The Netherlands; Amsterdam Reproduction and Development Research Institute, Amsterdam, The Netherlands; Department of Developmental Biology, Erasmus MC, Rotterdam, The Netherlands; Reproductive Biology Laboratory, Center for Reproductive Medicine, Amsterdam UMC, University of Amsterdam, Amsterdam, The Netherlands; Amsterdam Reproduction and Development Research Institute, Amsterdam, The Netherlands; Reproductive Biology Laboratory, Center for Reproductive Medicine, Amsterdam UMC, University of Amsterdam, Amsterdam, The Netherlands; Amsterdam Reproduction and Development Research Institute, Amsterdam, The Netherlands; Department of Anatomy and Embryology, Leiden University Medical Center, Leiden, The Netherlands; The Novo Nordisk Foundation Center for Stem Cell Medicine (reNEW), Leiden University Medical Center, Leiden, The Netherlands; Ghent-Fertility and Stem Cell Team (G-FAST), Department of Reproductive Medicine, Ghent University Hospital, Ghent, Belgium; Reproductive Biology Laboratory, Center for Reproductive Medicine, Amsterdam UMC, University of Amsterdam, Amsterdam, The Netherlands; Amsterdam Reproduction and Development Research Institute, Amsterdam, The Netherlands; Department of Developmental Biology, Erasmus MC, Rotterdam, The Netherlands

**Keywords:** pluripotent stem cells, gonadal development, somatic niche, Sertoli cells, granulosa cells, *in vitro* gametogenesis

## Abstract

**BACKGROUND:**

The gonadal somatic niche is crucial for sex determination and gamete formation throughout the human life cycle. However, key steps in gonadal somatic lineage differentiation occur during embryonic and foetal development, making them difficult to study in humans. *In vitro* differentiation models are therefore needed to investigate gonadal development, support *in vitro* gametogenesis, and study infertility. A comprehensive overview of gonadal somatic niche differentiation, both *in vivo* and *in vitro*, is thus crucial.

**OBJECTIVE AND RATIONALE:**

This review connects *in vivo* knowledge with *in vitro* differentiation systems for gonadal somatic niches, predominantly focusing on cell–cell signalling factors. It evaluates existing *in vitro* protocols for differentiating testicular and ovarian somatic niches, discusses them in the context of *in vivo* findings, and explores potential advancements in model systems.

**SEARCH METHODS:**

A narrative review was conducted after a comprehensive search of the PubMed database through to February 2025; the review focused on search topics including: *in vivo* gonadal differentiation in humans and mice; *in vitro* differentiation of human embryonic stem cells or human-induced pluripotent stem cells into gonadal somatic cells (bipotential, Sertoli or granulosa cells); and evidence for the cell–cell signalling factors used in these protocols.

**OUTCOMES:**

We investigated various strategies that aim to differentiate human pluripotent stem cells into gonadal somatic cell lineages. These include sequential growth factor differentiation recapitulating all known developmental progenitor stages, directed growth factor differentiation that omitted one or more developmental intermediates, and directed overexpression of key transcription factors. To induce differentiation, the growth factor-based protocols used various cell–cell signalling factors, with some derived from *in vivo* studies, while others lacked direct *in vivo* evidence. Despite significant advances in guiding pluripotent stem cells towards gonadal differentiation, challenges remain, such as the limited molecular and functional validation of the generated cell types. Consequently, complete human *in vitro* gametogenesis through co-culture techniques with pluripotent cell-derived germ cells has not yet been achieved, indicating that full functional maturation of the gonadal niche has not been attained with the current protocols.

**WIDER IMPLICATIONS:**

Integrating knowledge on *in vivo* gonadal development with enhanced differentiation protocols offers the potential to reliably generate the gonadal somatic niche *in vitro*. This allows for more accurate modelling of the gonad, facilitating deeper insights into the normal and pathological processes involved in gonadal development and germ cell maturation. For example, it could help to identify mechanisms linked to infertility or differences of sex development. Importantly, as many of these models are based on human pluripotent stem cells, they have the potential for personalization, enabling future patient-specific models for studying reproductive disorders and developing tailored fertility treatments.

**REGISTRATION NUMBER:**

n/a.

## Graphical abstract

**Figure:**
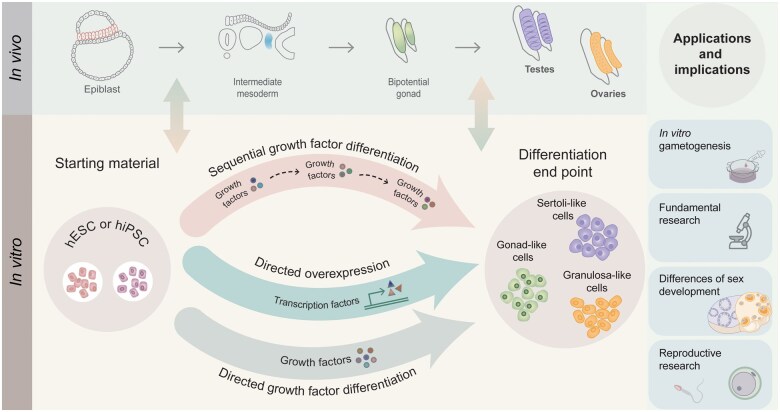
Optimizing human pluripotent stem cell-based gonadal niche models: sequential growth factor, directed growth factor and overexpression approaches informed by *in vivo* insights.

## Introduction

Germline stem cells, like all stem cells, depend on the somatic niche for their functional maintenance and differentiation. This specialized microenvironment begins to form in the embryo, preparing to receive and support the primordial germ cells (PGCs) that arise from a different embryonic lineage. Within this niche, PGCs are directed towards one of two mutually exclusive fates during sex determination. This process is guided by the differentiation of somatic progenitors into either Sertoli cells, which are essential for testis formation and spermatogenesis, or granulosa cells, which are necessary for ovarian follicle development and oogenesis (Richardson and Lehmann, 2010; Tang *et al.*, 2016; Rotgers *et al.*, 2018). Understanding the germ cell-somatic niche interplay is crucial for advancing reproductive health and for studying infertility and differences of sex development (DSD).

The scarcity of early human embryonic material has hindered gonadal development research, highlighting the need for developing representative models, such as *in vitro* gametogenesis (IVG) (Ishii and Saitou, 2017; Hong *et al.*, 2021). Despite advancements in human stem cell differentiation models, the gonadal niche remains challenging to recapitulate *in vitro* due to the complex signalling interactions involved in its development. This review primarily focuses on the current progress made in addressing this challenge.

A promising approach to study the influence of the somatic niche on germ cell development involves deriving both, germ cells and their supporting niche, from human embryonic or induced pluripotent stem cells (hESCs/hiPSCs, human pluripotent stem cells (hPSCs)), to recreate gametogenesis *in vitro* (Lan *et al.*, 2013; Kjartansdóttir *et al.*, 2015; Liu *et al.*, 2016; Sepponen *et al.*, 2017; Lipskind *et al.*, 2018; Rodríguez Gutiérrez *et al.*, 2018; Liang *et al.*, 2019; Knarston *et al.*, 2020; Hart *et al.*, 2022; Pryzhkova *et al.*, 2022; Gonen *et al.*, 2023; Pierson Smela *et al.*, 2023). Several protocols for deriving human PGC-like cells (hPGCLCs) from hiPSCs have been established (Overeem *et al.*, 2023; Esfahani *et al.*, 2024), and previously reviewed (Saitou and Hayashi, 2021; Teague *et al.*, 2023). Recent advances have also enabled hPGCLC expansion and their differentiation towards migratory and post-migratory stages (Alves-Lopes *et al.*, 2023; Murase *et al.*, 2024). However, a functional *in vitro* model of the human gonadal somatic niche is currently lacking. Consequently, attempts for human IVG have been conducted through co-culture systems of primary rodent somatic niche cells and hPGCLCs (Ishikura *et al.*, 2016; Yamashiro *et al.*, 2018; Hwang *et al.*, 2020). However, using non-human somatic cells for human IVG is suboptimal due to known and unknown interspecies differences in gene regulatory networks, associated with genetic and epigenetic differences, and it also comes with ethical and safety concerns. Consequently, recent IVG attempts have focussed on transitioning from rodent-derived soma to human-derived alternatives (Alves-Lopes *et al.*, 2023; Overeem *et al.*, 2023; Pierson Smela *et al.*, 2023), highlighting the need to generate a functional hPSC-derived somatic niche.

Evaluating and improving these *in vitro* human gonadal niche models necessitates a profound understanding of *in vivo* gonadal development and the processes harnessing them. Due to ethical constraints associated with human embryonic research, research to date on human gonadal niche development has primarily relied on histological investigations, *ex vivo* cultures of human foetal samples, and cases of DSD. Consequently, current human data are predominantly descriptive, with mechanistic knowledge mostly derived from animal models.

The aim of this review is to summarize the state-of-the-art *in vitro* models for hESC/hiPSC to Sertoli or granulosa cell differentiation, and evaluate the pathways recapitulated in these models by placing them in the context of *in vivo* development. Sertoli and granulosa cells are the focus of this review since they are the first sex-specific cell types formed in the gonad, guiding further differentiation of the testes and ovaries, respectively. The first section of this review will provide an overview of human *in vivo* gonadal niche development, starting with the formation of the bipotential gonad and progressing to sex-specific differentiation. It should be noted that there may be some variability in morphological and cell differentiation events in human gonadal development in relation to embryo development as a whole, which may explain why reported timing of developmental milestones may differ across studies. Still, for consistency, we use postconceptional weeks (PCWs) to denote gestational staging. By identifying knowledge gaps in human gonadal development and supplementing them with insights from animal and *ex vivo* models, a comprehensive perspective on the field is provided. The second section evaluates the existing *in vitro* protocols for deriving Sertoli and granulosa cells, drawing insights from our understanding of *in vivo* development. By exploring these aspects, we aim to contribute to the improvement of protocols that recreate the gonadal niche *in vitro*, ultimately advancing the understanding of reproductive biology.

## Methods

For this narrative review, a search of the PubMed database was conducted, targeting studies until 10 February 2025. The search for relevant gonadal somatic cell, Sertoli cell, or granulosa cell differentiation protocols included (combinations of) MeSH terms such as cell differentiation, stem cell differentiation, *in vitro* techniques, cellular reprogramming, human embryonic stem cells, induced pluripotent stem cells, gonads, Sertoli cells, granulosa cells, and human(s). The titles and abstracts of these studies were assessed for relevance, followed by full-text analysis. Protocols are described in this study if they were hESCs or hiPSCs based, used addition of growth factors (GFs) or transcription factor overexpression for differentiation, and were in English. Animal studies and differentiation protocols targeting gonadal lineages other than bipotential somatic progenitors, Sertoli or granulosa cells were deemed irrelevant. Additional searches were conducted through combination of search terms targeting aspects of *in vivo* gonadal development, focussing on the known morphological and cell state transitions between mesoderm and Sertoli or granulosa cell formation. This was complemented with a search for publications on the function and/or expression of marker genes and on the cell–cell signalling factors used in the relevant protocols. Subsequently, relevant studies were analysed to provide an in-depth overview of the current state of research in this area.

## Human gonadal development *in vivo*: addressing the knowledge gaps for *in vitro* modelling

**Figure 1.**
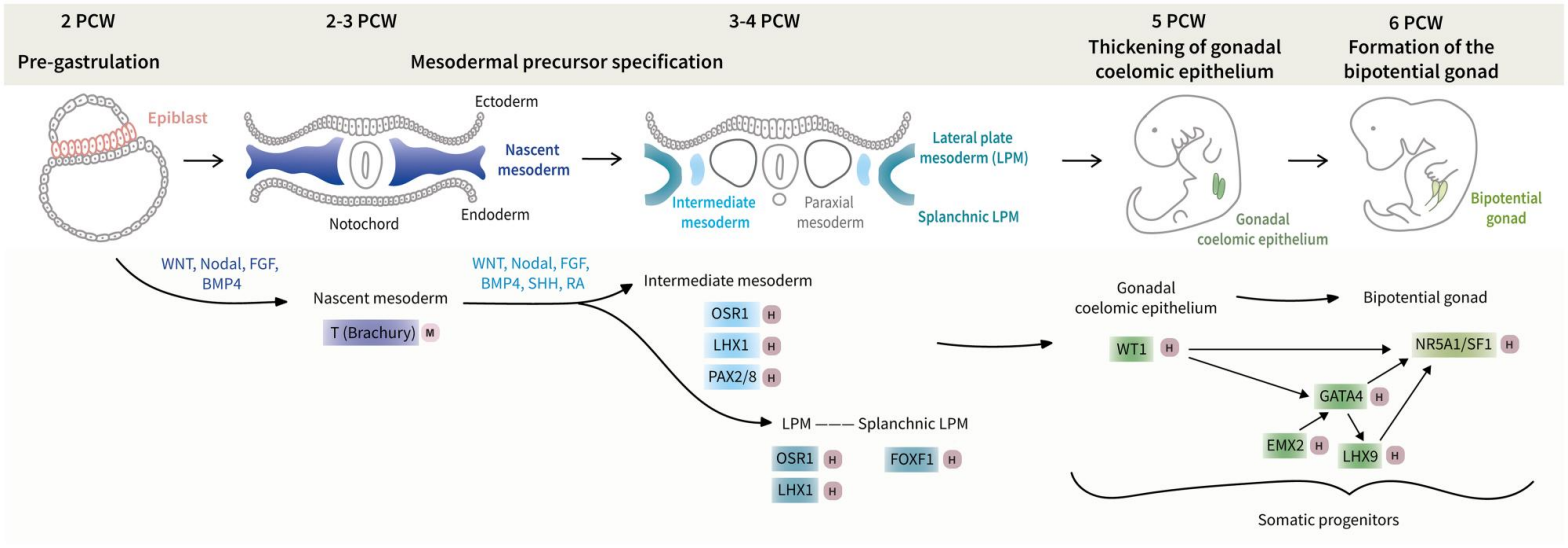
**Schematic representation of the differentiation trajectory from the human epiblast to the bipotential gonad, highlighting the sequential differentiation of embryonic tissue progenitors**. The upper panel indicates the developmental stages along the timeline in postconceptional weeks (PCWs). In the middle panel, progenitor cell types that contribute to the bipotential gonad are indicated, colour-coded by cell type, and shown with their location within the embryo (schematics for up to 3–4 PCWs represent transverse sections, while five and six PCW schematics represent whole embryos). The lower panel shows key signalling and transcription factors known to be involved in these differentiation processes. Black arrows indicate activating interactions, with signalling factors labelled above the arrows, and transcription factors shown in coloured boxes corresponding to each cell type. Light/dark pink squares beside transcription factors indicate the type of evidence, with ‘M’ denoting evidence from mouse studies and ‘H’ denoting evidence from human studies, or that both types of evidence are present.

**Figure 2.**
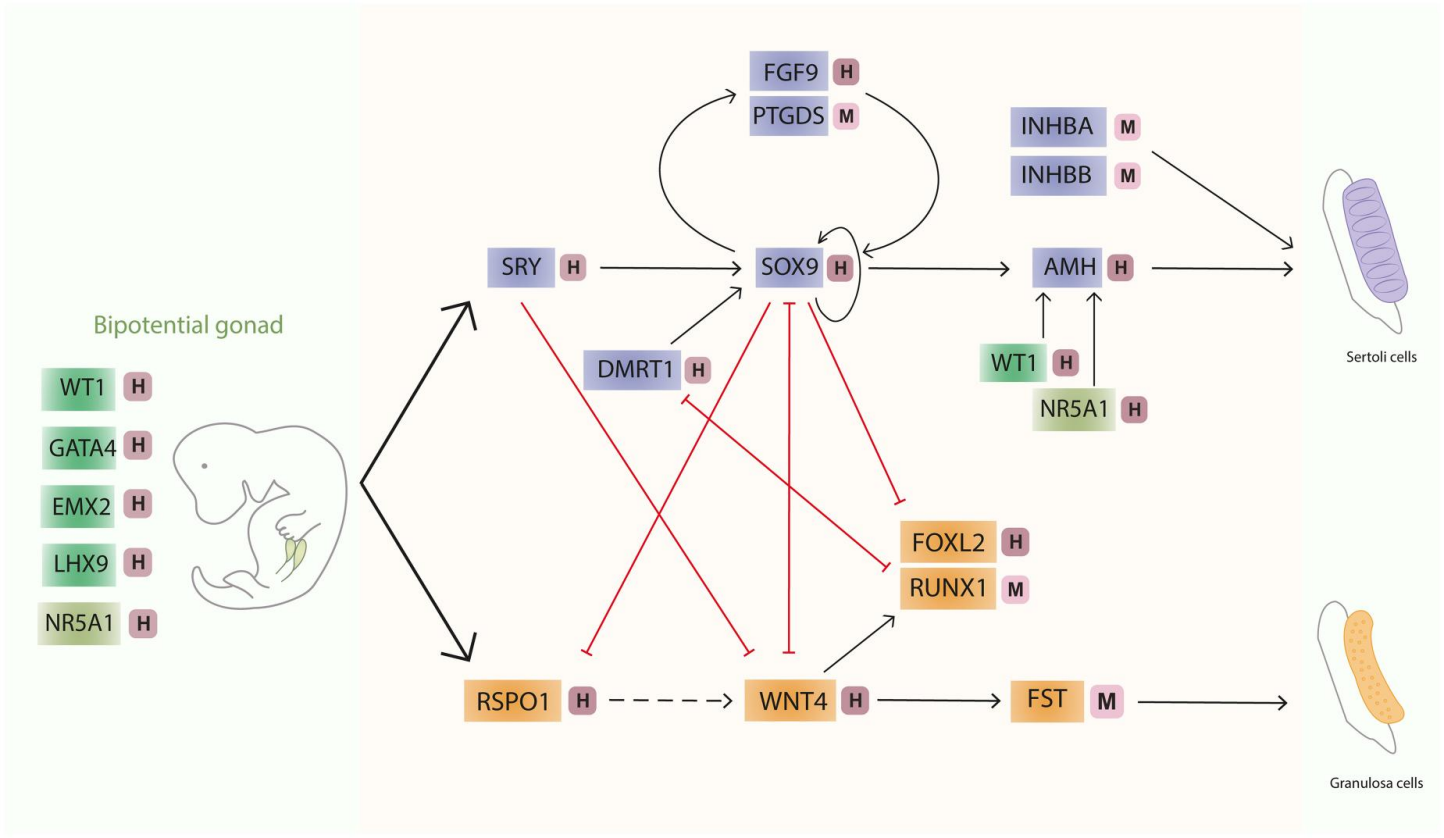
**Regulatory network directing bipotential gonadal progenitors into Sertoli cells (testicular soma) or granulosa cells (ovarian soma), including antagonistic interactions contributing to maintenance of the specified fates throughout development and adulthood**. This schematic figure illustrates the proteins that act in a (gene) regulatory network crucial for the specification of the bipotential gonad cells (green) into Sertoli cell fate (purple) and granulosa cell fate (orange). Black arrows indicate activating regulation (either direct or through activation of gene expression), dashed black arrows indicate indirect activation, curved black arrows represent positive feedback loops, while red lines denote either negative regulation or mutual antagonism between factors. Light/dark pink squares beside the genes indicate the type of evidence, with ‘M’ denoting evidence from mouse studies and ‘H’ denoting evidence from human studies, or that both types of evidence are present.

Gonadogenesis in mammals occurs in two distinct stages: bipotential and sex-specific stages. The bipotential stage involves gonadal progenitor cell specification within the bipotential gonad (Fig. 1), whereas the sex-specific stage comprises the subsequent differentiation of bipotential gonadal progenitors into either the testicular or ovarian supporting cells, depending on the genetic sex of the individual (Fig. 2).

### Gonadal mesodermal precursors

The onset of human gonadal differentiation depends on mesodermal precursor specification during gastrulation, leading to nascent mesoderm formation between 2 and 3 PCWs (Fig. 1, ‘Mesodermal precursor specification’) (O’Rahilly and Müller, 1987; Tyser *et al.*, 2021; Cui *et al.*, 2025). Critical gastrulation regulators in humans are unknown, but in mice, nascent mesoderm differentiates under the influence of Wingless-related integration site (WNT), transforming growth factor (TGF)-beta/Nodal, bone morphogenetic protein (BMP), and fibroblast growth factor (FGF) signalling pathways (Winnier *et al.*, 1995; Liu *et al.*, 1999; Ciruna and Rossant, 2001; Ben-Haim *et al.*, 2006). The intermediate mesoderm (IM), one of the mesodermal subtypes, forms by the end of the third PCW in humans (Fig. 1, ‘Mesodermal precursor specification’) (O’Rahilly and Müller, 1987; Zeng *et al.*, 2023). The IM is thought to be the urogenital ridge progenitor, including the pronephros, metanephros, mesonephros, and the gonads. Marker genes *OSR1*, *LHX1*, and *PAX2/8* are expressed in the IM during 3–4 PCWs (Xu *et al.*, 2023; Zeng *et al.*, 2023). Mutations or deletions in these genes in humans have been associated with developmental abnormalities of the kidneys and genital ducts (Martinovic-Bouriel *et al.*, 2010; Zhang *et al.*, 2011; Ledig *et al.*, 2012a; Lozić *et al.*, 2014; Rasmussen *et al.*, 2016). However, their necessity for human gonadal differentiation is unclear, as gonadal development was not investigated in these individuals. In mice, *OSR1* and *LHX1* knockout models display gonadal agenesis, whereas *PAX2/8* double knockout mice exhibit normal gonads (Shawlot and Behringer, 1995; Bouchard *et al.*, 2002; Wang *et al.*, 2005). This highlights that not all known IM marker genes are essential for gonadal development in humans and mice.

Despite the lack of evidence for other mesodermal lineages contributing to human gonadogenesis, recent mouse transcriptomic studies have suggested that a subtype of lateral plate mesoderm (LPM), the splanchnic LPM, is a more probable gonadal precursor than the IM (Fig. 1, ‘Mesodermal precursor specification’) (Neirijnck *et al.*, 2023; Qiu *et al.*, 2024). However, due to the embryonic lethality of splanchnic LPM marker gene knockouts, these *in silico* lineage tracing predictions remain unverified experimentally (Firulli *et al.*, 1998; Mahlapuu *et al.*, 2001). The distinction between IM and LPM is challenging due to their proximity within the embryo and conflicting reports regarding marker gene expression. Studies of third PCW embryos showed varying results: one observed *OSR1* and *LHX1* expression in both IM and LPM (Zeng *et al.*, 2023), while another found no *OSR1* expression in LPM (Xiao *et al.*, 2024). These discrepancies may be due to differences in morphological staging or sequencing depth between studies. Nonetheless, immunofluorescence studies in human and mouse embryos have shown that gonadal progenitors are located next to cells expressing FOXF1, a splanchnic LPM-specific marker, but do not express FOXF1 themselves (Sasaki *et al.*, 2021; Cheng *et al.*, 2022). This suggests that gonadal progenitors either downregulate *FOXF1* upon differentiation or do not originate from splanchnic LPM. Another complicating factor is that both IM and LPM undergo differentiation regulated by complex gradients of signalling factors along various embryonic axes. These include Nodal and BMP4 along the medio-lateral axis, sonic hedgehog and BMP4 along the dorso-ventral axis, and WNT, retinoic acid (RA), and FGF along the anterior–posterior axis (Tonegawa *et al.*, 1997; Niederreither *et al.*, 1999; Sakai *et al.*, 2001; James and Schultheiss, 2005; Sweetman *et al.*, 2008; Fleming *et al.*, 2013; Kumar and Duester, 2014; Ariza *et al.*, 2016). The observed similarities between IM and LPM, and the diverse morphogen gradients involved in their differentiation, present challenges in delineating which subtype of mesoderm needs to be recapitulated during *in vitro* differentiation.

### Emergence of the genital ridge

The precise morphological and molecular events following IM or LPM specification, leading to subsequent gonadal progenitor formation remain poorly understood, and the identity of this progenitor in humans is debated. Recent morphological and transcriptomic studies proposed the coelomic epithelium (CE) as the likely precursor (Fig. 1, ‘Thickening of gonadal coelomic epithelium’) (Garcia-Alonso *et al.*, 2022; Himelreich Perić *et al.*, 2023), while earlier research suggested the mesonephros, or both CE and mesonephros (Wartenberg, 1978, 1982; Satoh, 1991). The CE, which overlies the IM and the mesonephros, exhibits epithelial marker gene expression in humans (Cheng *et al.*, 2022). During the third PCW, both IM and mesonephros already express the transcription factor WT1, while the CE acquires WT1 expression around this time, as this expression is extended in ventrolateral and anterior-to-posterior directions along the CE (Cheng *et al.*, 2022). These molecular changes are followed by a morphological transformation: between 4 and 5 PCWs, the CE thickens, and the genital ridge becomes visible (Wartenberg, 1982; Satoh, 1991; Cheng *et al.*, 2022; Himelreich Perić *et al.*, 2023). A mechanistic understanding on this subject has been established in mice, where both CE and mesonephric cells have been shown to migrate into the gonad, contributing to somatic cell lineages, although the functional contribution of mesonephric cells to the mouse testes has been disputed (Martineau *et al.*, 1997; Karl and Capel, 1998; Cunha *et al.*, 2023). Additionally, the initial CE thickening was demonstrated to rely on WNT signalling and involve asymmetric cell division at the basolateral CE domain in mice (Chassot *et al.*, 2012; Lin *et al.*, 2017). Thus, the CE remains the most probable genital ridge progenitor.

### Somatic progenitor differentiation in the bipotential gonad

The transcription factors guiding differentiation of CE (and/or mesonephros) into gonadal somatic progenitors have been extensively investigated (Fig. 1, ‘Formation of the bipotential gonad’). Immunofluorescence studies revealed that WT1-positive cells within the human genital ridge initiate GATA4 expression during the fourth PCW, followed by NR5A1 and LHX9 expression early in the fifth PCW (Cheng *et al.*, 2022). Another study observed co-expression of *WT1* and *NR5A1* in cells within the human genital ridge midway through the fourth PCW through RNA *in situ* hybridization (Hanley *et al.*, 1999). Additionally, expression of the transcription factor EMX2 has been reported in human XY gonads at the protein level during the fifth PCW (Ostrer *et al.*, 2007), and in human XX gonads post-sex determination in scRNA-seq data (Garcia-Alonso *et al.*, 2022). Mutations or deletions in *WT1*, *GATA4*, *LHX9*, *NR5A1*, and *EMX2* have been linked to various human cases of DSD, presenting with phenotypes ranging from (partial) gonadal dysgenesis, intersex conditions, sex reversal, and primary ovarian insufficiency (Pelletier *et al.*, 1991; Köhler *et al.*, 2008, 2011; Lourenço *et al.*, 2009, 2011; Piard *et al.*, 2014; Bashamboo *et al.*, 2016; Hoefele *et al.*, 2017; Wang *et al.*, 2018; Eozenou *et al.*, 2020; Kunitomo *et al.*, 2020; Ferrari *et al.*, 2022). These phenotypes support the role of WT1, GATA4, LHX9, NR5A1, and EMX2 in bipotential gonad development as well as sex determination in humans.

Regulatory interactions between these identified key transcription factors are derived from functional mouse studies (Fig. 1, ‘Formation of the bipotential gonad’) (Birk *et al.*, 2000; Wilhelm and Englert, 2002; Kusaka *et al.*, 2010; Hu *et al.*, 2013). Knowledge of cell–cell signalling factors involved in gonadal somatic progenitor differentiation is limited. However, insulin-like GF signalling has been implicated in the proliferation of both, GATA4 and NR5A1 positive gonadal progenitor cells in mice (Pitetti *et al.*, 2013). Once specified, gonadal progenitors expressing WT1, GATA4, NR5A1, and LHX9 are believed to differentiate into gonadal somatic cells (XY or XX).

### Sex specification within the gonad

The sex-specific stage of gonadal differentiation in humans initiates around 5–6 PCWs when the gonadal bipotential progenitor cells begin to specify into either testicular or ovarian somatic lineages (Fig. 2). Understanding the regulatory mechanisms that underlie this process is crucial for developing and refining *in vitro* culture systems that recapitulate gonadal development.

#### Molecular mechanisms driving testicular fate determination

In the bipotential gonad of an XY embryo, the onset of SRY expression triggers a signalling cascade that directs the gonadal progenitors towards a Sertoli cell fate. In human embryos, SRY is first detected around the sixth PCW, and gradually declines in expression between 8 and 9 PCWs (Hanley *et al.*, 2000; Mamsen *et al.*, 2017). Functional studies in mice have shown that SRY activates transcription of *SOX9*, encoding a transcription factor that promotes Sertoli cell differentiation, while actively suppressing the ovarian fate (Fig. 2) (Sekido *et al.*, 2004; Wilhelm *et al.*, 2005). Additional research in mice has shown that SRY acts together with NR5A1 to initiate *SOX9* expression. Subsequently, SOX9 autonomously sustains its own expression (Sekido and Lovell-Badge, 2008). In accordance, deletions or duplications in the promoter or enhancer regions of *SOX9* have been linked to complete sex reversal and malformations in external genitalia or monorchidism, respectively, in humans with DSD (Benko *et al.*, 2011; Lybæk *et al.*, 2014; Kim *et al.*, 2015; Gonen *et al.*, 2018; Croft *et al.*, 2018). Another key factor that enhances *SOX9* expression in humans is DMRT1 (Fig. 2) (Raymond *et al.*, 1999; Jørgensen *et al.*, 2012). *DMRT1* serves as a sex determination or maintenance gene in Sertoli cells, as its mutations in 46, XY individuals cause sex reversal with female genitalia, and either partial or complete gonadal dysgenesis (Flejter *et al.*, 1998; Raymond *et al.*, 1999; Ledig *et al.*, 2012b; Chauhan *et al.*, 2017).

Around the seventh PCW, the essential autocrine/paracrine signalling regulator FGF9 is first detected (Ostrer *et al.*, 2007) and further antagonizes the ovarian fate (Fig. 2) (Kim *et al.*, 2006). Human DSD studies support this regulatory control, as evidenced by a 46, XY individual with mutated *FGF9*, who presented with female internal and external genitalia, and degenerated streak gonads (Croft *et al.*, 2023). Similarly, a 46,XX individual with *FGF9* duplication presented with atrophied testes and azoospermia, thereby indicating the role of this gene in actively antagonizing ovarian specification (Chiang *et al.*, 2013). Moreover, *ex vivo* cultures of foetal testes demonstrated that FGF9 stimulated Sertoli cell proliferation and was essential for maintaining SOX9 and anti-Müllerian hormone (AMH) expression levels (Harpelunde Poulsen *et al.*, 2019). An additional critical gene activated by SOX9 in mice is *PTGDS*, which encodes the enzyme Prostaglandin D2 (PGD2) synthase. In humans, the role of PGD2, which is produced from the eighth PCW, is not well documented except in the context of human foetal testes exposure to endocrine disruptors (Mazaud-Guittot *et al.*, 2013; Ben Maamar *et al.*, 2017). However, PGD2 signalling in mice contributes to the feedforward loop sustaining *SOX9* expression, similar to the FGF9-*SOX9* feedforward loop (Fig. 2) (Wilhelm *et al.*, 2005; Moniot *et al.*, 2009). Initial *PTGDS* activation occurs independent of *FGF9*, but both feedforward loops are required to maintain sufficient SOX9 levels for testis differentiation (Wilhelm *et al.*, 2005; Moniot *et al.*, 2009). Considered collectively, adequate control and stabilization of SOX9 expression are crucial in the differentiation and maintenance of Sertoli cells, and should be achieved in XY *in vitro* systems.

#### Molecular dynamics post Sertoli cell fate determination

Following Sertoli cell fate establishment, testicular cord formation is initiated coinciding with the secretion of one of the earliest Sertoli cell markers, AMH, typically detected around 7–8 PCWs (Ostrer *et al.*, 2007). *In vitro* studies using promotor-reporter constructs in different cell lines have demonstrated that transcription factors SOX9, WT1, and NR5A1 activate *AMH* transcription (Fig. 2) (De Santa Barbara *et al.*, 1998; Nachtigal *et al.*, 1998), which is crucial for Müllerian duct regression (Guerrier *et al.*, 1989). Other TGF-beta family members, including activins and inhibins, have been identified in murine models as potential contributors to Sertoli cell differentiation (Sarraj *et al.*, 2010; Miles *et al.*, 2013). Activin A (*INHBA*), a specific ligand within this family, has been implicated in Sertoli cell proliferation, an essential event occurring between 8 and 16 PCWs in human development (Ostrer *et al.*, 2007; Archambeault and Yao, 2010; Nicholls *et al.*, 2012; Jørgensen and Rajpert-De Meyts, 2014). Furthermore, Activin B (*INHBB*) has been shown to regulate testicular cord organization in mice (Yao *et al.*, 2006). Although testis formation is not affected in *AMH* knockout mice (Behringer *et al.*, 1994), *AMH* and *INHBB* double knockout models exhibited a failure to maintain Sertoli cell identity, with partial transdifferentiation into granulosa cells, leading to incomplete sex reversal (Rodriguez *et al.*, 2022). Additionally, inhibition of Activin and Nodal signalling in *ex vivo* human foetal testis culture resulted in decreased AMH expression and disrupted seminiferous cord morphology (Jorgensen *et al.*, 2018). Together, these findings highlight the crucial role of AMH and TGF-beta signalling networks in Sertoli cell differentiation and testicular cord formation.

Sertoli cell differentiation coordinates testicular morphogenesis, thereby compartmentalizing the niche into regions for spermatogenesis (testicular cords, surrounded by peritubular myoid cells) and steroidogenesis (interstitium, with Leydig cell development) (Cool *et al.*, 2012; Stévant *et al.*, 2018, 2019; Guo *et al.*, 2021). Development of these testis-specific cell types is directed by Sertoli cells, and is not discussed in this review. Further functional development of the human testis involves resumed proliferation of the Sertoli cell population during early post-natal life, followed by a quiescent period and subsequent proliferation during puberty (Cortes *et al.*, 1987; Sharpe *et al.*, 2003). However, upon puberty, human Sertoli cells cease proliferation and become terminally differentiated, a key feature of mature Sertoli cells. This is accompanied by upregulated expression of the androgen receptor (*AR*), a mature Sertoli cell marker, while AMH expression is downregulated (Boukari *et al.*, 2009; Lapoirie *et al.*, 2021). Changes in expression pattern and the interplay of several other regulators upon initiation of puberty trigger the formation of Sertoli cell junctions that eventually form the blood–testis barrier. Despite extensive research, the mechanisms driving final Sertoli cell maturation and accompanying alterations in the gene expression profile remain elusive in both mouse and human, thereby challenging development of *in vitro* protocols for these final steps in Sertoli cell maturation.

#### Molecular mechanisms of ovarian fate specification

Ovarian differentiation is a complex process influenced by a variety of transcription and signalling factors, steering away from the Sertoli cell differentiation pathway. The first wave of pre-granulosa cell formation arises around the seventh PCW in humans, likely originating from bipotential somatic progenitors in the medulla of the foetal ovary (Wartenberg, 1982; Mamsen *et al.*, 2017; Garcia-Alonso *et al.*, 2022; Lardenois *et al.*, 2023; Wamaitha *et al.*, 2023). During this developmental stage, human embryonic ovaries express *WNT4* and R-spondin 1 (*RSPO1*), with RSPO1 enhancing WNT signalling (Fig. 2). (Tomaselli *et al.*, 2011). Both genes play a role in granulosa cell differentiation, as mutations in *WNT4* and *RSPO1* have been associated with sex reversal in individuals with DSD (Jordan *et al.*, 2001; Parma *et al.*, 2006; Mandel *et al.*, 2008). Mouse studies have revealed that WNT4 fulfils this role through antagonism of *FGF9* (Vainio *et al.*, 1999; Kim *et al.*, 2006). Consistent with this, findings from *ex vivo* foetal testis and ovary cultures demonstrated that WNT stimulation disrupted seminiferous cord formation and downregulated SOX9 expression, while ovarian tissues were unaffected (Lundgaard Riis *et al.*, 2024). This highlights that ovarian differentiation is an active process involving suppression of male differentiation.

Further mechanistic research in mice demonstrated that WNT4 and RSPO1 cooperatively promote granulosa cell differentiation through induction of Follistatin (*FST*), an activin inhibiting factor, and transcription factor *FOXL2* gene expression (Fig. 2) (Maatouk *et al.*, 2008; Tomizuka *et al.*, 2008; Tomaselli *et al.*, 2011; Li *et al.*, 2017b). In humans, *FOXL2* also serves as an early granulosa cell marker and *FOXL2* mutations have been associated with premature ovarian failure (Zlotogora *et al.*, 1983; Duffin *et al.*, 2009; Garcia-Alonso *et al.*, 2022). The mutually exclusive expression of FOXL2 and SOX9 in human foetal gonads suggests SOX9-FOXL2 antagonism during development (Hersmus *et al.*, 2008). Studies in mice have confirmed this antagonistic action, as well as the involvement of *FOXL2* in granulosa cell maintenance (Ottolenghi *et al.*, 2005; Uhlenhaut *et al.*, 2009; Li *et al.*, 2017b).

During mouse granulosa cell maintenance, the transcription factor RUNX1 plays a complementary role to FOXL2 (Nicol *et al.*, 2019). While no *RUNX1* mutations have been connected to DSD or infertility in humans, higher expression has been documented in XX gonads compared to XY gonads from the sixth PCW onwards (Nicol *et al.*, 2019). However, these results were not recapitulated in scRNA-seq data (Wamaitha *et al.*, 2023). Nonetheless, it is evident that the principal pathways governing granulosa cell differentiation are conserved between humans and mice.

By the eighth PCW, a second wave of pre-granulosa cell formation appears in the human ovarian cortex, likely originating from the ovarian surface epithelium (Garcia-Alonso *et al.*, 2022; Wamaitha *et al.*, 2023). These cells are characterized by lower levels of *FOXL2* expression and a less prominent WNT signalling signature compared to the first wave (Garcia-Alonso *et al.*, 2022; Wamaitha *et al.*, 2023). The functional significance of the two waves of pre-granulosa cell formation remains uncertain, and it is thus unclear whether both waves need to be recapitulated during *in vitro* granulosa cell differentiation protocols.

Ovarian stromal cells arise from a presumably common progenitor to the pre-granulosa cells (Lardenois *et al.*, 2023; Wamaitha *et al.*, 2023). They infiltrate the human ovary between 6 and 7 PCWs, surround the cord-like structures formed by pre-granulosa cells, and are transcriptionally similar to testicular interstitial cells (Satoh, 1991; Motta *et al.*, 1997; Wamaitha *et al.*, 2023). While this compartment may be relevant for *in vitro* gonadal niche models, it falls outside the scope of this review.

#### Post-natal ovarian development and follicle maturation

During post-natal and pubertal development, granulosa cells within the follicle differentiate into two distinct types: cumulus and mural cells. Cumulus cells form transzonal projections to maintain contact with the oocyte, whereas mural cells line the periphery of the follicle (Albertini *et al.*, 2001). The oocyte-secreted signalling factors GDF9 and BMP15 play a crucial role in cumulus cell specification, as mutations in the genes encoding these factors are associated with conditions like primary amenorrhea and premature ovarian failure in humans (Laissue *et al.*, 2006; Rossetti *et al.*, 2009). FSH is also involved in human post-natal granulosa cell differentiation, as demonstrated by cases of isolated FSH deficiency leading to arrested follicle development at the primordial follicle stage (Rabin *et al.*, 1972). Additionally, human growth hormone (HGH) has been implicated in post-natal granulosa cell differentiation, as HGH treatment in IVF patients increased the density of FSH receptors (FSHR) and LH/choriogonadotropin receptors (Regan *et al.* 2018). These signalling factors could thus be applied *in vitro* to modulate the post-natal granulosa cell fate.

During further follicle development, as multiple layers of granulosa cells are formed, the steroid-producing theca cells also differentiate and form the outer cell layer of the so-called secondary follicle (not addressed further in this review). Once follicles mature into the antral stage, the LH surge results in cumulus cell expansion and ovulation (Russell and Robker, 2007). This subdivision of granulosa cell type and function during follicular development should also be recapitulated when modelling follicle maturation *in vitro*.

## *In vitro* modelling of the foetal human somatic niche: current protocols and their correlation with *in vivo* gonadal development

**Figure 3.**
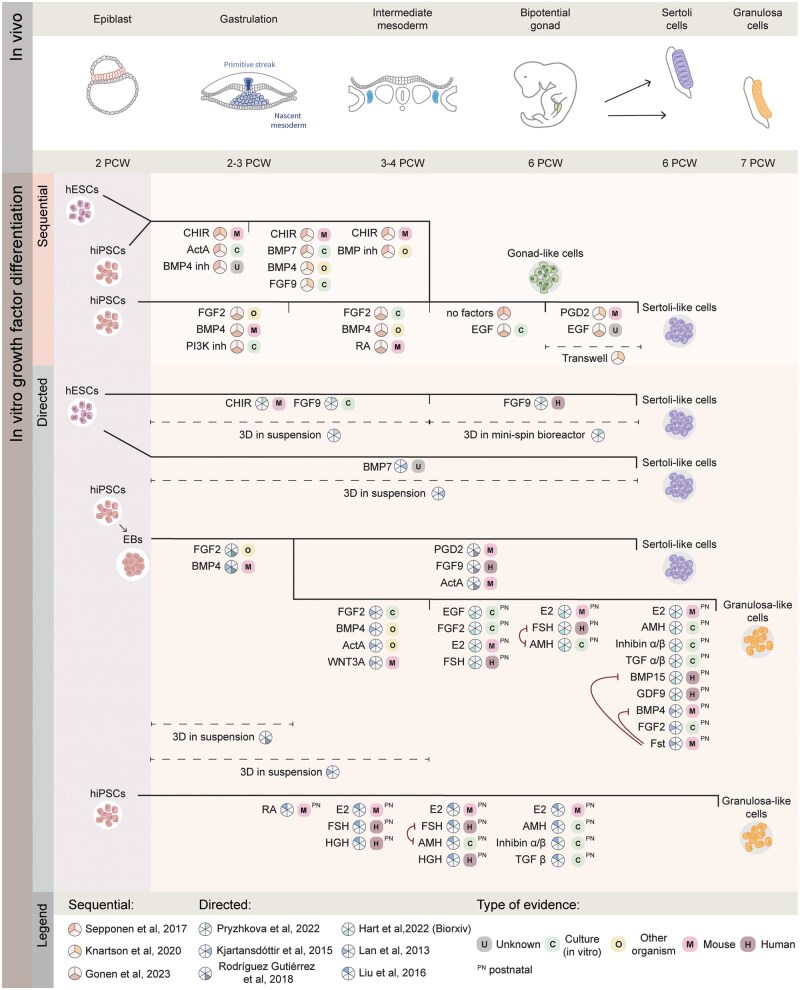
**Overview of human *in vitro* gonadal somatic niche differentiation protocols that make use of growth factors**. The upper section shows the corresponding developmental stages *in vivo*. Within the *in vitro* section, the solid black lines with vertical dashes represent these stages. The grey circles with schematic cell representations show protocol end points. The coloured pie charts indicate which protocols used the factor, with the references indicated in the legend. The coloured ‘squares’ indicate the type of evidence found for a certain growth factor, as indicated in Supplementary Table S1. The type of evidence is ordered based on relevance from left (low) to right (high) in the legend, with only the most relevant type displayed. Post-natal (PN) indicates that the factor only plays a role in post-natal development of the somatic niche. The black dashed lines indicate periods of culture using 3D culture methods, with the coloured pie chart indicating which protocol used these methods. The dark purple lines indicate evidence for inhibitory action of one factor on another *in vivo*.

**Figure 4.**
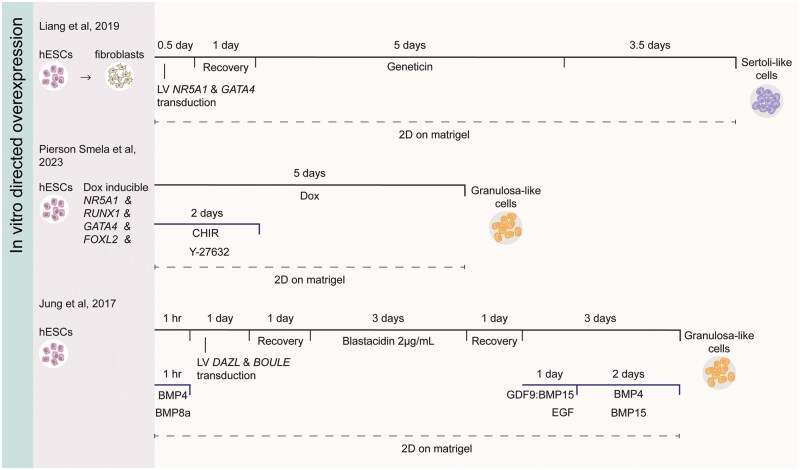
**Overview of directed transcription factor overexpression protocols for human *in vitro* gonadal somatic niche differentiation**. The left panel indicates the reference and starting material for each protocol. Solid black lines represent protocol duration (above) and gene expression manipulations (e.g. lentiviral transduction, antibiotic selection, doxycycline induction) (below). Dark grey lines denote growth factor supplementation duration (above) and specific factors added (below). The ‘:’ between GDF9 and BMP15 indicates that a protein heterodimer was supplemented. Dashed black lines indicate the periods of culture using a culture method (2D/3D) and substrate. Grey circles with schematic cell representations mark protocol endpoints. Dox, doxycycline; LV, lentiviral.

Leveraging insights from *in vivo* developmental knowledge enables the development of *in vitro* differentiation protocols to generate human Sertoli-like cells (hSLCs) and granulosa-like cells (hGLCs). This section will focus on relevant hPSCs to hSLC or hGLC differentiation protocols. Most of these studies have relied on growth factor administration to drive cell fate modifications *in vitro* (Tables 1 and 2: ‘Sequential growth factor’ and ‘Directed growth factor’, Fig. 3) (Lan *et al.*, 2013; Kjartansdóttir *et al.*, 2015; Liu *et al.*, 2016; Sepponen *et al.*, 2017; Rodríguez Gutiérrez *et al.*, 2018; Knarston *et al.*, 2020; Hart *et al.*, 2022; Pryzhkova *et al.*, 2022; Gonen *et al.*, 2023), while a few used transcription factor overexpression approaches (Tables 1 and 2, ‘Directed overexpression’, Fig. 4) (Jung *et al.*, 2017; Liang *et al.*, 2019; Pierson Smela *et al.*, 2023). The sequential growth factor differentiation follows the known *in vivo* developmental stages and thus requires monitoring of progression through each successive differentiation step. In contrast, the directed growth factor differentiation and transcription factor overexpression streamline the process by omitting one or more of these stages, potentially offering a faster route to generate the desired cell type, but also holding a risk of only partially recapitulating the transcriptional signature of the desired cell type.

**Table 1.** Overview of *in vitro* differentiation protocols towards human Sertoli-like cells.

	Reference	Starting material	ECS/iPSC maintenance medium	Differentiation medium		Developmental stage					
						*Gastrulation*	*Intermediate mesoderm*		*Bipotential gonad*	*Sertoli cell*	
Sequential growth factor	Sepponen *et al.* (2017)	ESC	Essential 8	DMEM: F12 1:1 + Glutamax + 1x B27	Substrate	2D on collagen type I					
					Duration	*8 days*					
					Prot L	*1 day*	*2 days*	*1 day*	*4 days*		
					Growth factors	CHIR (5 μM) Activin A (100 ng/ml) Dorsomorphin (2 µM) Y-27632 (10 μM)	CHIR (3 μM) for 24 h BMP7 (10 ng/ml) for 48 h	Dorsomorphin (2 μM)	No growth factors		
					Prot M	1 day	1 day	2 days	4 days		
					Growth factors	CHIR (5 μM) Activin A (100 ng/ml) Dorsomorphin (2 μM) Y-27632 (10 μM)	CHIR (3 μM) BMP7 (50 ng/ml)	CHIR (3 μM) Dorsomorphin (2 μM)	No growth factors		
					Associated markers	*T*		*OSR1* *LHX1* *PAX2*	*WT1 &* **WT1** *GATA4 &* **GATA4** *LHX9* *EMX2*		
	Knarston *et al.* (2020)	iPSC	Essential 8	Essential 6 or DMEM: F12 1:1 + 0.54 mg/ml sodium bicarbonate + 14 ng/ml sodium selenite + 64 μg/ml AA+ 10 μg/ml Holo-Transferrin + 20 μg/ml insulin	Substrate	2D on vitronectin or Matrigel				Transwell filter	
					Duration	*21 days*					
					Growth factors	*4 days*	*3 days*		*3 days*	*11 days*	
						CHIR (3 μM)	BMP4 (10 ng/ml) FGF9 (200 ng/ml) Heparin (1 μg/ml)		No growth factors	PDG2 (500 ng/ml)	
					Associated markers		*OSR1* *LHX1* *PAX2*		*WT1 &* **WT1** *GATA4 &* **GATA4** *LHX9* *EMX2* *NR5A1* *GADD45G* *ZFPM2* *HSD3B2 &* **3BHS2**	*SOX9 &* **SOX9** *AMH &* **AMH** *FGF9* *CLDN11 &* **CLDN11** *DHH*	
Sequential growth factor	Gonen *et al.* (2023)	iPSC	mTESRplus	Days 0–3.5: Advanced DMEM: IMDM 1:1 + 0.1% polyvinyl alcohol + 1% P/S + 1:100 concentrated lipids + 4 μM monothioglycerol + 17 μg/ml transferrin	Substrate	2D on Matrigel					
					Duration	*12 days*					
					Growth factors	*1.5 days*	*2 days*		*2 days*	*6.5 days*	
						FGF2 (20 ng/ml) Ly294002 (10 μM) BMP4 (10 ng/m)	FGF2 (5 ng/ml) BMP4 (20 ng/ml) RA (100 nM)		EGF (20 ng/ml)	EGF (20 ng/ml)	
				Days 3.5–12: Advanced DMEM+ 1% P/S + 1:100 ITS	Associated markers	*T*	*OSR1*		*WT1* *GATA4* *NR5A1*	*SOX9 &* **SOX9** **AMH** *FGF9* *DMRT1* **CLDN11**	
Directed growth factor	Pryzhkova *et al.* (2022)	ESC aggregated to EB	Essential 8	Days 0–1: Essential 8	Substrate	3D (suspension)				3D in spin bioreactor	
				Days 1–40: E8 + AA + 2-phosphate sesquimagnesium salt hydrate + 1x ITS + 100 U/100 ug P/S+ FGF2	Duration	*40 days*					
					Growth factors	*N/A*	*3 days*	*3 days*	*N/A*	*Up to 34 days*	
						N/A	CHIR (8 μM) for 72 h Y-27632 (5 μM) for 24 h	FGF9 (10 ng/ml)	N/A	FGF9 (10 ng/ml)	
					Associated markers					Days 16–20: **GATA4**	Days 20–40: **SOX9**
										Days 16–40: **WT1** **AMH** **INHB** Day 26, 35/36: **SRY** **DMRT1**	Days 10–40: *WT1* *LHX9* *NR5A1* *SIX1/4* *SRY* *SOX9*
Directed growth factor	Kjartansdóttir *et al.* (2015)	ESC	Adherent: KO-DMEM + 20% KOSR + 0.5% P/S + 1% NEAA + 2 mM L-glutamax + 0.5 mM 2-mercaptoethanol + 8 ng/ml rFGF2	Adherent: KO-DMEM + 20% KOSR + 0.5% P/S + 1% NEAA + 2 mM L-glutamax + 0.5 2-mercaptoethanol	Substrate	2D (on mitotically inactivated human foreskin fibroblasts) or 3D (suspension)					
					Duration	*14 days*					
					Growth factors	BMP7 (10 ng/ml)					
					Associated markers	N/A	N/A	N/A	N/A	**WT1** **SOX9** **AMH** *VIM* & **VIM** *FSHR* After 48 h hCG (5 IU) and FSH (5 IU) stimulation: **Inhibin B**	
			Sphere aggregation: Neurobasal + 14% KOSR + 2 mM Glutamax + 0.5% P/S + 1% NEAA + 20 ng/ml rFGF2 + 25 ng/ml Activin A + 1 μg/ml fibronectin + 0.5 μg/ml human placental laminin + 0.001% porcine gelatin + 10 ng/ml rBDNF + 10 ng/ml rNT3 + 10 ng/ml rNT4 + 1x Nutridoma	Suspension: KO-DMEM: F12 1:1							
	Rodríguez Gutiérrez *et al.* (2018)	iPSC aggregated to EB	F12 + 10% KOSR + 5% NEAA + 1% 2-Mercaptoethanol+ 5% P/S + 10 ng/ml FGF2	EB aggregation and Days 0–1: F12 + 10% KOSR + 5% NEAA + 1% 2-Mercaptoethanol+ 5% P/S	Substrate	3D	EB attachment culture on NT2d1 cells and matrigel				
					Duration	*13 days*					
						*1 day*				*Up to 12 days*	
					Growth factors	FGF2 (50 ng/ml) BMP4 (30 ng/ml)	N/A	N/A	N/A	FGF9 (50 ng/ml) PGD2 (500 ng/ml) Activin A (40 ng/ml)	
				Days 1–12: Medium A (unspecified)							
					Associated markers					*WT1* *GATA4* *SOX9 &* **SOX9** *FGF9* *SRY* *AMH &* **AMH** *INHBB* *CYP26B1* *HSD17B1* *CLDN11 &* **CLDN11**	*DHH* *AR* *VIM* *NR0B1* *PTGDS* *SRC* *KRT18* *FSHR*
Directed growth factor	Liang *et al.* (2019)	ESC- differentiated to fibroblasts LV *GATA4/NR5A1* overexpression	ES state: KODMEM + 20% KOSR + 1 mM l-glutamine + 0.1 mM NEAA + 4 ng/ml FGF2	DMEM + 10% FBS + 1 mM l-glutamine + 0.1 mM NEAA	Substrate					2D on Matrigel	
					Duration	*N/A*				*10 days*	
						N/A				*6.5 days*	*3.5 days*
										LV transduction	
										LV supernatant 12 h Recovery 1 day Geneticin (1 mg/ml) 5 days	
			ES differentiated to fibroblasts: KODMEM + 20% KOSR + 1 mM l-glutamine + 0.1 mM NEAA		Associated markers	N/A					*SOX9* **AMH** *KRT18 &* **KRT18** *PTGDS* *CDKN1B* *CLU*
					Functional readout						48 h of sustained mouse spermatogonial culture

Sequential growth factor, directed growth factor, and overexpression protocols to derive human Sertoli-like cells are summarized, outlining different methodological parameters. Left panel indicates the protocol type. Only the final optimized protocol is included when optimizations were reported, with growth factors listed for the duration of their use and the transcription factors selected for overexpression listed in the starting material column. Associated markers in italics indicate qPCR and bulk RNA-seq results, associated markers in bold indicate immunofluorescence, flow cytometry, or secreted hormones. Only analysed marker genes relevant to the developmental stages are included, with others omitted. Functional readouts refer to supporting germ cell survival or maturation. AA, ascorbic acid; DMEM, Dulbecco’s Modified Eagle’s Medium; EB, embryoid body; IMDM, Iscove Modified Dulbecco Medium; ITS, insulin transferrin selenium; KOSR, knock-out serum replacement; KO DMEM, Knockout DMEM; LV, lentiviral; NEAA, non-essential amino acids; P/S, Penicillin–Streptomycin; Prot, protocol.

**Table 2.** Overview of *in vitro* differentiation protocols towards human granulosa-like cells.

	Reference	Starting material	ESC/iPSC maintenance medium	Differentiation medium		Developmental stage								
						*Gastrulation*	*Intermediate mesoderm*	*Granulosa*						
Directed growth factor	Hart *et al.* (2022)	iPSC aggregated to EB	mTESR1	DMEM: F12 1:1 +	Substrate	EB attachment culture on Matrigel								
				10% KSOR + 5% NEAA + 1% 2-Mercaptoethanol + 5% P/S + 10 ng/ml FGF2	Duration	*12 days*								
					Growth factors	*1 day*	*N/A*	*2 days*	*2 days*		*4 days*			
						FGF2 (50 ng/ml) BMP4 (30 ng/ml)	N/A	EGF (20 ng/ml) FGF2 (50 ng/ml) E2 (20 ng/ml) FSH (20 ng/ml)	E2 (20 ng/ml) FSH (20 ng/ml) AMH (20 ng/ml)		Protocol A: E2 (20 ng/ml) AMH (20 ng/ml) Inhibin-alpha (20 ng/ml) Inhibin-beta (20 ng/ml) TGF-beta (15 ng/ml) TGF-alpha (15 ng/ml)			Protocol B: E2 (20 ng/ml) AMH (20 ng/ml) Inhibin alpha (20 ng/ml) Inhibin beta (20 ng/ml) BMP15 (25 ng/ml) GDF9 (50 ng/ml)
				Long term culture: DMEM: F12 + 2% FCS + 1% NEAA + 0.05 μM dex + 100 mM L-glutamine + 0.5% P/S + 20 ng/ml EGF + 50 ng/ml FGF2 + 20 ng/ml FSH	Associated markers			*FOXL2 &* **FOXL2** *FSHR &* **FSHR** *INHBA* *AMH &* **AMH** *STAR* *ZEB2* *CD44* *CYP11A1* *CYP19A1* *HSD3B2* *HSD17B1* *NR5A1* *AR* *ESR1*						*TST* *KITLG* *CDCA3* *NRG1* *PGR* *FST* *STAR* *HSPG2* *KDSR* *CHST8* *ESR2* *PGR* *LHR* **E2**
	Lan *et al.* (2013)	iPSC aggregated to EB	Primate embryonic stem medium + 4 ng/ml FGF2	EB aggregation and differentiation: DMEM: F12 8:2 + 20% KOSR + 0.1 mM 2-mercaptoethanol + 1 % NEAA + 4 ng/ml FGF2	Substrate	EB suspension culture		EB attachment culture on gelatin						
					Duration	*10 days*								
						*1 day*	*3 days*	*6 days*						
					Growth factors	BMP4 (5 ng/ml)	FGF2 (5 ng/ml) BMP4 (10 ng/ml) Activin A (6 ng/ml) WNT3A (6 ng/ml)	BMP4 (10 ng/ml) FGF2 (5 ng/ml) FST (25 ng/ml)						
					Associated markers	*T* & **T** *GSC*	*OSR1* *PAX2*						*FOXL2* *FSHR &* **FSHR** *AMH &* **AMH** *LHR* *CYP19A1 &* **CYP19A1** *AMHR2 &* **AMHR2** **E2**	
Directed growth factor	Liu *et al.* (2016)	iPSC	DMEM: F12 1:1 + 15% KOSR + 1 mM sodium pyruvate + 2 mM L-glutamine + 0.1 mM NEAA + 0.1 mM 2-mercaptoethanol + 25 U/ml 925 mg/ml P/S + 15 ng/ml FGF2 + 15 ng/ml EGF	Unspecified	Substrate	2D								
					Duration	*12 days*								
					Growth factors	*N/A* N/A	*N/A*	*2 days*	*2 days*	*2 days*	*4 days*			*2 days*
							N/A	RA (15 ng/ml)	E2 (20 ng/ml) FSH (20 ng/ml) HGH (10 ng/ml)	E2 (20 ng/ml) FSH (20 ng/ml) AMH (20 ng/ml) HGH (10 ng/ml)	E2 (20 ng/ml) AMH (20 ng/ml) Inhibin-alpha (20 ng/ml) Inhibin-beta (20 ng/ml) TGF-beta (15 ng/ml)			No factors
					Associated markers									**AMH** **FSHR** **Inhibin-alpha** **Inhibin-beta**
					Functional readout									Reduced number of atretic follicles in PCOS mouse model
Directed overexpression	Pierson Smela *et al.* (2023)	iPSC— Epi *NR5A1/* *RUNX1/* *GATA4/* *FOXL2* overexpression	mTESRplus	DMEM: F12 + 10% KOSR + 15 mM HEPES + 1x Glutamax	Substrate	2D on Matrigel								
					Duration	*5 days*								
						*2 days*	*3 days*							
						Doxycycline (gene induction) (1 µg/ml)	Doxycycline (gene induction) (1 µg/ml)							
					Growth factors	CHIR (3 μM) Y-27632 (10 µM)	N/A							
					Associated markers							*AMHR2* **CD82** **E2** **P4**		
					Functional readout							70 days of sustained hPGCLC co-culture		
	Jung *et al.* (2017)	ESC— LV *DAZL/* *BOULE* overexpression	KODMEM + 20% KOSR + 1 mM l-glutamine + 0.1 mM NEAA + 8 ng/ml FGF2	KODMEM + 10% FBS + 1 mM l-glutamine + 0.1 mM NEAA	Substrate	2D on Matrigel								
					Duration	9 days								
					Growth factors	N/A N/A	N/A N/A	*2 days*	*4 days*		*1 day*			*2 days*
								LV transduction	Blasticidin selection (2 μg/ml) for 3 days					
								1 h BMP4 (50 ng/ml) 1 h BMP8a (50 ng/ml) 24 h LV supernatant			GDF9: BMP15 (0.35 ng/ml) EGF (10 ng/ml)			GDF9 (50 ng/ml) BMP15 (25 ng/ml)
					Associated markers									*RSPO1* **AMH ** *INHBA* *INHBB* *LGR5* *AREG* *HAS2* *LHX8* *LHX9* *CYP19A*
					Functional readout									Follicle formation when transplanted under mouse kidney capsule

Directed growth factor and overexpression-based protocols to derive human granulosa-like cells are summarized, outlining different methodological parameters. Left panel indicates the protocol type. Only the final optimized protocol is included when optimizations were reported, with growth factors and pharmaceutical compounds listed for the duration of their use and the transcription factors selected for overexpression listed in the starting material column. Associated markers in italics indicate qPCR and bulk/sc-RNA-seq results, associated markers in bold indicate immunofluorescence, flow cytometry, or secreted hormones. Only analysed marker genes relevant to the developmental stages are included, with others omitted. Functional readouts refer to supporting germ cell survival or maturation. AA, ascorbic acid; DMEM, Dulbecco’s Modified Eagle’s Medium; EB, embryoid body; Epi, episomal; E2, Estradiol; ITS, insulin transferrin selenium; KOSR, Knockout serum replacement; KO DMEM, Knockout DMEM; LV, lentiviral; NEAA, non-essential amino acids; P/S, Penicillin–Streptomycin; KOSR, knock-out serum replacement; P4, Progesterone.

This section analyses 12 hSLCs and hGLCs differentiation protocols, comparing their starting materials, culture conditions, and experimental readouts within the framework of *in vivo* developmental knowledge. Tables 1 and 2 provide comprehensive overviews of available protocols for hSLC (seven protocols) and hGLC (five protocols) differentiation, respectively. Figure 3 schematically illustrates the nine growth factor-based protocols, highlighting commonly used growth factors, their supportive evidence, and the implementation of 3D culture methods. Figure 4 schematically depicts the three directed overexpression protocols. Supplementary Table S1 summarizes the evidence supporting the use of growth factors shown in Fig. 3.

### Choice of pluripotent stem cells and their maintenance media

Several primary parameters other than differentiation methodology can influence the success and outcomes of the differentiation protocols. The choice of hESCs or hiPSCs as the starting material may necessitate specific optimization due to their distinct epigenetic landscapes and the influence of the hiPSC reprogramming methods (Nichols and Smith, 2009; Takahashi *et al.*, 2018). In addition, only 3 out of the 12 analysed studies used the same culture medium (Essential 8) for stem cell maintenance (Tables 1 and 2, ‘ESC/iPSC maintenance medium’). Differences between the maintenance media could influence differentiation outcomes due to differences in the metabolic state or genetic and epigenetic stability of the hPSCs prior to differentiation (Garitaonandia *et al.*, 2015; Prakash Bangalore *et al.*, 2017). Finally, cell line-specific genetic and epigenetic differences may also account for some of the observed differences in marker gene induction among studies. The number of cell lines used in the analysed studies ranged from one to four, with only one study evaluating the influence of different cell lines on differentiation outcomes (Kjartansdóttir *et al.*, 2015). Their analyses revealed that, although the culturing method (adherent vs 3D in suspension) had the greatest influence on the transcriptomic profiles during maintenance culture, the three analysed cell lines displayed varying degrees of marker gene induction during differentiation. Consequently, the differences discussed in the following sections warrant careful interpretation within the context of the starting material, cell line, and culture methods employed.

### *In vitro* modelling of gastrulation through modulation of signalling pathways

Six (four hSLC and two hGLC) out of nine growth factor-based studies (six hSLC and three hGLC, Tables 1 and 2, ‘Sequential growth factor’ and ‘Directed growth factor’) initiated differentiation by inducing a mesoderm-like fate *in vitro*. The terminology for this state varied, with studies referring to it as either ‘primitive streak’ (Sepponen *et al.*, 2017; Knarston *et al.*, 2020) or ‘mesoderm’ (Lan *et al.*, 2013; Rodríguez Gutiérrez *et al.*, 2018; Hart *et al.*, 2022; Gonen *et al.*, 2023). Three of these six studies provided no explicit verification of this cell fate (two hSLC and one hGLC), while the other three evaluated *T* as a marker gene (two hSLC, one hGLC), irrespective of the terminology used (Tables 1 and 2, column ‘Gastrulation’). Lan *et al.* (2013, hSLC) assessed an additional marker gene, *GSC* and reported its expression during the gastrulation-like differentiation step.

In mice, WNT and BMP signals are well-established regulators of early gastrulation and mesodermal specification (Supplementary Table S1) (Takada *et al.*, 1994; Winnier *et al.*, 1995; Liu *et al.*, 1999; Yamaguchi *et al.*, 1999). Accordingly, two out of the six protocols induced a gastrulation-like fate through activation of WNT signalling by administering CHIR, a glycogen synthase kinase-3 inhibitor, that functions as a WNT agonist (two hSLC, Fig. 3) (Sepponen *et al.*, 2017; Knarston *et al.*, 2020). Four out of the six protocols employed BMP stimulation for gastrulation-like differentiation (two hSLC and two hGLC, Fig. 3), (Lan *et al.*, 2013; Rodríguez Gutiérrez *et al.*, 2018; Hart *et al.*, 2022; Gonen *et al.*, 2023), while one combined activation of WNT with inhibition of the BMP pathway (one hSLC, Fig. 3) (Sepponen *et al.*, 2017). Notably, none of the studies combined WNT and BMP stimulation at this stage, unlike the protocol for foetal mouse ovarian somatic-like cell differentiation (Yoshino *et al.*, 2021). This divergence could reflect species-specific or pluripotent state-specific differences between mESC and hPSCs (Nichols and Smith, 2009). Mechanistically, BMP signalling has been shown to induce the WNT pathway during hESC differentiation, possibly rendering either pathway sufficient for inducing a gastrulation-like fate *in vitro* (Kurek *et al.*, 2015).

FGF and activin signalling, alongside WNT and BMP factors, are commonly used to induce a gastrulation-like fate from hPSCs *in vitro* (Supplementary Table S1) (Wang and Chen, 2016). FGF2 was administered during this differentiation stage in three out of six studies aiming for a gastrulation-like fate (two hSLC and one hGLC, Fig. 3, ‘Gastrulation’) (Rodríguez Gutiérrez *et al.*, 2018; Hart *et al.*, 2022; Gonen *et al.*, 2023), while one incorporated Activin A instead (one hSLC, Fig. 3, ‘Gastrulation’) (Sepponen *et al.*, 2017). Neither FGF2 nor Activin A has been implicated in rodent or human gastrulation *in vivo*, despite their involvement in mesoderm specification in other species (Supplementary Table S1) (Asashima *et al.*, 1990; Smith *et al.*, 1990; Kimelman and Maas, 1992; Hug *et al.*, 1997; Nutt *et al.*, 2001). This lack of evidence for FGF- or Activin A-dependent mammalian mesoderm differentiation *in vivo* might be due to functional redundancy among factors that share receptors or downstream effects within the signalling networks (Supplementary Table S1) (Song *et al.*, 1999; Ben-Haim *et al.*, 2006).

*In vitro* studies support this hypothesis and have shown that FGF2 is essential for BMP-dependent mesoderm differentiation of hPSCs (Bernardo *et al.*, 2011; Yu *et al.*, 2011). Likewise, downstream factors of Activin A signalling have been linked to direct induction of mesodermal marker genes, including *T* in hPSCs RNA-seq and ChIP-seq studies (Wang and Chen, 2016). Notably, using these factors in differentiation protocols requires consideration of their roles in proliferation, pluripotency maintenance, and self-renewal during routine hPSC culture (Beattie *et al.*, 2005; Xu *et al.*, 2005; Bertero *et al.*, 2015). One model has suggested that FGF2 alone maintains the hPSC undifferentiated state, while its combination with BMP and WNT promotes a mesendodermal fate (Wang and Chen, 2016). Conversely, Activin A requires PI3K pathway inhibition to induce differentiation in hPSCs (Singh *et al.*, 2012).

Overall, FGF2 with BMP4 was the most used growth factor combination for mesoderm induction, supported by *in vivo* and *in vitro* evidence. However, most protocols confirmed gastrulation fate by analysing only a single marker gene, *T*, also induced by WNT and Activin A. Therefore, multiple marker genes should be analysed during future optimizations, combined with modulating multiple signalling pathways by optimizing growth factor combinations and concentrations, rather than relying on individual GFs.

### *In vitro* modulation of mesodermal fate post-gastrulation

Five out of the nine growth factor-based differentiation protocols aimed for an IM-like fate induction either following gastrulation-like differentiation (three hSLC and one hGLC, Tables 1 and 2, ‘Sequential growth factor’ and ‘Directed growth factor’) or directly from the hESC state (one hSLC, Table 1, ‘Directed growth factor’) (Lan *et al.*, 2013; Sepponen *et al.*, 2017; Knarston *et al.*, 2020; Pryzhkova *et al.*, 2022; Gonen *et al.*, 2023). Four of these studies confirmed IM fate induction through measurement of *OSR1* expression (three hSLC and one hGLC, Tables 1 and 2), three assessed *PAX2* (two hSLC and one hGLC, Tables 1 and 2), and two measured *LHX1* (two hSLC Table 1). The IM differentiation stage showed greater variability in growth factors used across protocols than the gastrulation-like stage (Fig. 3). This variability may reflect differences in obtained cell states following gastrulation-like differentiation, resulting in varied signalling requirements, or varying efforts to mimic mesodermal specialization along the anteroposterior and mediolateral axes of the embryo (Fowler *et al.*, 2020). Despite methodological differences, all studies investigating IM marker gene expression observed induction at the appropriate stage (Lan *et al.*, 2013; Sepponen *et al.*, 2017; Knarston *et al.*, 2020; Gonen *et al.*, 2023).

As described in the *in vivo* section, WNT and RA signalling are well-established morphogenic regulators of the mesodermal fate along the anteroposterior axis in mice (Fig. 1) (Niederreither *et al.*, 1999; Sweetman *et al.*, 2008; Kumar and Duester, 2014). *In vitro*, differentiation along this axis can be mimicked by addition of these factors, and fine-tuned by varying concentration and duration. For instance, longer exposure to WNT signalling during mesoderm differentiation has been associated with the differentiation of more posterior IM-derived cell types (Taguchi *et al.*, 2014; Takasato *et al.*, 2015; Taguchi and Nishinakamura, 2017). Two of the studies analysed here investigated the influence of WNT pulse duration on IM differentiation outcomes, although neither assessed the expression of anterior or posterior IM marker genes (two hSLC, Table 1, ‘Sequential growth factor’) (Sepponen *et al.*, 2017; Knarston *et al.*, 2020). Sepponen *et al.* (2017) found that stimulation with CHIR for 2–4 days during mesodermal differentiation stages led to the highest expression of gonadal marker genes in the subsequent stages. Similarly, Knarston *et al.* (2020) identified a 4-day exposure to CHIR at this stage as optimal.

Conversely, the anteriorizing factor RA was used in only one hSLC differentiation study during IM induction (Fig. 3, ‘Intermediate mesoderm’) (Gonen *et al.*, 2023). While this study did not directly compare hiPSC differentiation outcomes with and without RA, the parallel mESC experiments demonstrated that RA treatment, combined with FGF2 and BMP4, effectively induced both IM and bipotential gonad marker genes. This finding aligns with a hPSC to renal lineage differentiation study in which the combination of RA and FGF2 treatment led to the induction of LHX1 and PAX2 double-positive cells with an efficiency of 70–80% (Lam *et al.*, 2014).

All three protocols that induced WNT signalling during IM differentiation utilized prolonged exposure (2–4 days), suggesting that a posterior IM fate was optimal for *in vitro* bipotential gonad-like cell induction. However, the IM cells generated by the RA, FGF2, and BMP4 combination require further analysis to determine whether these exhibited a similar or more anterior IM identity.

In addition to WNT and RA signalling, either FGF2 (one hSLC and one hGLC) (Lan *et al.*, 2013; Gonen *et al.*, 2023) or FGF9 (two hSLC) (Knarston *et al.*, 2020; Pryzhkova *et al.*, 2022) were incorporated into four out of the five studies aiming for IM differentiation (Fig. 3, ‘Intermediate mesoderm’). However, none of these studies compared differentiation outcomes with or without FGF stimulation. One possibility is that FGF2 or FGF9 directly influence IM differentiation. However, mouse knock-out models of these factors do not display defects in gonadal formation, indicating that they might not be essential (Dono *et al.*, 1998; Barak *et al.*, 2012). *In vitro*, FGF2 or FGF9, but not FGF8, was able to induce IM marker gene expression in the context of hiPSC differentiation into the renal lineage (Takasato *et al.*, 2014). Alternatively, FGF2 or FGF9 might mimic other FGF factors (FGF4/8) by binding to the same receptors *in vitro* (Hui *et al.*, 2018). In mice, *FGF4* and *FGF8* are involved in axis elongation and posterior somite differentiation (Boulet and Capecchi, 2012). Hence, modulating FGF signalling during IM specification *in vitro* could induce IM marker gene expression or stimulate a posterior fate during differentiation, again supporting the posterior IM as the optimal gonadal progenitor *in vitro*.

BMP signalling, a regulator of mesodermal fate along the mediolateral axis *in vivo* (Fig. 1) (Tonegawa *et al.*, 1997; James and Schultheiss, 2005), was utilized in four out of five IM-targeted protocols (three hSLC and one hGLC, Fig. 3, ‘Intermediate mesoderm’) (Lan *et al.*, 2013; Sepponen *et al.*, 2017; Knarston *et al.*, 2020; Gonen *et al.*, 2023). However, only two studies measured the effects by comparing IM and LPM marker gene expression (Sepponen *et al.*, 2017; Knarston *et al.*, 2020). Sepponen *et al.* (2017) conducted a comparative analysis of hESCs treated with BMP2, BMP4 or BMP7. Their results demonstrated that BMP7 induced a bipotential gonad-like fate, whereas BMP4 promoted an LPM-like fate, based on marker gene expression. Furthermore, this study showed that LPM marker gene expression increased upon stimulation with higher concentrations of BMP7. Complementing these findings, Knarston *et al.* (2020) demonstrated that higher concentrations of BMP4 *in vitro* led to increased expression of LPM marker genes (Knarston *et al.*, 2020). In conclusion, higher concentrations of BMP signalling *in vitro* promote the modelling of a more lateral mesodermal fate. However, the optimal intermediate differentiation stage for gonadal lineage specification remains uncertain, with ongoing debate if the progenitor should be IM, (splanchnic) LPM, or a combination of both.

As described in the section on *in vivo* gonadal development, IM is postulated to give rise to gonadal, renal, and adrenal cell types (Cheng *et al.*, 2022). Consequently, optimization of growth factor concentrations and their temporal supplementation is crucial to mimic the specific signalling environment promoting gonadal development while avoiding renal or adrenal differentiation. Nonetheless, only two of the analysed studies (two hSLC, Table 1, ‘Sequential growth factor’ and ‘Directed growth factor’) evaluated the formation of non-gonadal urogenital tissues *in vitro* (Knarston *et al.*, 2020; Pryzhkova *et al.*, 2022). Knarston *et al.* (2020) found no or minimal induction of adrenal (*SULT2A1*, *ARHGAP36*) or renal (*NPHS2*) marker genes during differentiation, while Pryzhkova *et al.* (2022) observed the formation of mesonephric tubule-like structures in their bioreactor-based 3D model. Due to this scarcity of information, optimal growth factor conditions for gonadal lineage progenitor differentiation, while avoiding other lineages, remain undetermined.

Despite uncertainty regarding the exact mesodermal gonadal progenitor, two studies evaluated both IM and LPM marker gene expression during hPSC differentiation towards bipotential gonad-like cells (Sepponen *et al.*, 2017; Knarston *et al.*, 2020). Sepponen *et al.* (2017) concluded that lower expression of the LPM marker HAND1 correlated with a higher percentage of GATA4 positive cells during differentiation, indicating bipotential gonad identity. Knarston *et al.* (2020) observed that the LPM marker gene expression differentially influenced gonadal marker genes. A more lateral mesodermal fate enhanced the induction of *GADD45G*, *GATA4*, *NR0B1*, *HSD3B2*, and *SOX9*, while a more medial fate increased the expression of *WT1*, *NR5A1*, and *EMX2*. However, interpreting the *in vitro* mesodermal marker expression requires caution due to overlapping expression patterns of genes across different mesodermal subtypes, as discussed in the *in vivo* section. Most studies incorporated the lateralizing factor BMP4 during IM differentiation. However, *GATA4* was the only gene analysed by multiple studies in this context, and conflicting results make it difficult to determine the optimal degree of mesoderm lateralization for gonadal differentiation.

### EGF is the only documented factor used for differentiation of bipotential gonad-like cells from a mesodermal progenitor

Three protocols described a sequential growth factor approach by inducing bipotential gonad-like cell differentiation following IM induction (three hSLC, Table 1, column ‘Bipotential gonad’) (Sepponen *et al.*, 2017; Knarston *et al.*, 2020; Gonen *et al.*, 2023). Two of these protocols did not add any growth factors after initial IM induction (Fig. 3, ‘Bipotential gonad’), but did observe an increase in *WT1*, *GATA4*, and *LHX9* expression over time, possibly explained by autocrine or paracrine signalling (Sepponen *et al.*, 2017; Knarston *et al.*, 2020). The third sequential growth factor protocol administered EGF after IM differentiation (Fig. 3, ‘Bipotential gonad’), and observed an increased expression of gonadal marker genes *GATA4* and *NR5A1*, concurrent with downregulated WT1 expression in a 46, XY human cell line (Gonen *et al.*, 2023). The precise effects of EGF stimulation on bipotential gonad-like cell differentiation remain elusive, primarily due to methodological limitations in existing studies. Gonen *et al.* (2023) did not include a comparative analysis between EGF-stimulated and non-stimulated conditions, hindering a direct assessment of the effects of EGF. Additionally, the *NR5A1* induction during the bipotential gonad-like stage upon EGF stimulation observed by Gonen *et al.* (2023), was similar to the *NR5A1* induction reported by Knarston *et al.* (2020) during air membrane interphase culture without any growth factors. However, the different reference genes used for normalization in these studies complicate direct comparison of *NR5A1* mRNA levels, underscoring the need for comparative analyses on the role of EGF in directing bipotential gonad-like cell differentiation.

Human gonadal transcriptomic studies performed between 6 and 21 PCWs have not specifically examined *EGF* or *EGFR* expression (Guo *et al.*, 2021; Garcia-Alonso *et al.*, 2022; Lardenois *et al.*, 2023; Wamaitha *et al.*, 2023). However, expression of both genes has been reported in human foetal ovaries between 10 and 22 PCWs, suggesting a potential developmental role (Yeh *et al.*, 1993). Yet, *EGF* knock-out mouse models are fertile and display normal testis morphology on E18 (Luetteke *et al.*, 1999; Levine *et al.*, 2000), indicating that EGF alone may not be essential for gonadal development. Possible functional redundancy among GFs activating similar downstream pathways, such as MAPK, might explain the lack of phenotypes in single-gene knock-out models (Wells, 1999; Tsang and Dawid, 2004). Nonetheless, EGF was also employed in the differentiation protocol of mouse foetal ovarian somatic-like cells (Yoshino *et al.*, 2021), suggesting a beneficial effect of EGF in gonadal *in vitro* differentiation strategies.

### Sequential growth factor induction of the Sertoli-like cell fate has been partly established

Two protocols described subsequent differentiation towards hSLCs after the bipotential gonad-like stage (Table 1, ‘Sequential growth factor’) (Knarston *et al.*, 2020; Gonen *et al.*, 2023). Knarston *et al.* (2020) generated hSLCs by exposing bipotential gonad-like cells to PGD2 (Fig. 3, ‘Sertoli’), a known Sertoli cell inducer in mice, as described in the *in vivo* section. Exposure to PGD2 resulted in the upregulation of *SOX9*, *FGF9*, *CLDN11*, and *AMH* in the derived hSLCs, with even higher induction of *SOX9*, *CDH11*, and *AMH* when using air–liquid interphase membrane culture compared to monolayer differentiation. Additionally, hSLCs grown on these membranes displayed tubular-like clusters of SOX9 and GATA4 positive cells, hinting at enhanced intrinsic organization compared to monolayer cultures. Alternatively, Gonen *et al.* (2023) exposed human bipotential gonad-like cells to EGF (Fig. 3, ‘Sertoli’), observing persistent expression of *SOX9* and AMH secretion for up to 7 weeks in culture. Differentiated XY cells in this study exhibited coordinated migration and formation of tubular-like structures when plated in a microfluidics device, unlike XX cells and XY DSD cells. Differences in culture methods and readouts between the two studies complicate recommendations on the optimal growth factors for the final hSLC differentiation step. Moreover, neither study validated whether the generated hSLCs can support germ cell maturation, which would be the key criterion for protocol selection. Furthermore, no study has described a sequential growth factor differentiation towards hGLCs after bipotential gonad-like fate induction. Consequently, the differentiation of functional hSLCs or hGLCs able to support germ cell maturation, through a sequential growth factor approach has not yet been achieved.

### Directed growth factor approaches drive differentiation into foetal Sertoli-like cells and post-natal granulosa-like cells

The second main approach for differentiating hSLCs or hGLCs involved directed growth factor differentiation, bypassing one or more of the *in vivo* developmental stages. This approach was described in six protocols (three hSLC and three hGLC, Tables 1 and 2, ‘Directed growth factor’) (Lan *et al.*, 2013; Kjartansdóttir *et al.*, 2015; Liu *et al.*, 2016; Rodríguez Gutiérrez *et al.*, 2018; Hart *et al.*, 2022; Pryzhkova *et al.*, 2022).

The initial differentiation steps differed between the three directed growth factor hSLC protocols (Table 1, ‘Directed growth factor’) (Kjartansdóttir *et al.*, 2015; Rodríguez Gutiérrez *et al.*, 2018; Pryzhkova *et al.*, 2022). Rodríguez Gutiérrez *et al.* (2018) initiated hiPSC differentiation by inducing a gastrulation-like state in embryoid bodies, followed by hSLC differentiation. Kjartansdóttir *et al.* (2015) aimed for direct hSLC differentiation from hESCs, while Pryzhkova *et al.* (2022) induced an IM fate from the hESC state before hSLC induction. During hSLC induction, two of these protocols used FGF9 (Fig. 3, ‘Directed’), a known factor involved in human Sertoli cell differentiation (Fig. 2). Pryzhkova *et al.* (2022) used FGF9 as the sole growth factor following IM specification, while Rodríguez Gutiérrez *et al.* (2018) combined FGF9 with PGD2 and Activin A, murine Sertoli cell inducing factors described in the *in vivo* section. In contrast, Kjartansdóttir *et al.* (2015) opted for a distinct approach using BMP7 alone during hESC to hSLC differentiation (Fig. 3, ‘Sertoli’).

All three studies reported the appearance of elongated or flattened morphology within the hSLC population and observed induction of SOX9 and AMH at the protein level (three hSLC, Table 1, ‘Directed growth factor’), although the SOX9 expression observed by Kjartansdóttir *et al.* (2015) was not nuclear. Additionally, Rodríguez Gutiérrez *et al.* (2018) characterized the transcriptomic signature of their hSLCs through bulk RNA sequencing, observing similar or higher Sertoli cell marker gene expression compared to a Sertoli cell line. Pryzhkova *et al.* (2022) characterized hSLC protein expression and observed that SOX9 was mutually exclusive with WT1, and AMH was present in the cytoplasm of only a minority of SOX9-positive cells. Moreover, two of these studies evaluated hSLC hormone secretion, with Rodríguez Gutiérrez *et al.* (2018) detecting AMH secretion in the medium, while Kjartansdóttir *et al.* (2015) found no evidence of Inhibin-beta secretion. None of the directed growth factor hSLC differentiation protocols have conclusively shown that the achieved molecular profile matches that of *in vivo* Sertoli cells. However, FGF9 was most commonly used during directed growth factor hSLC differentiation and serves as a promising starting point for future protocol optimization. Additionally, PGD2 has shown potential in both sequential and directed GF hSLC differentiation.

One of these directed growth factor hESC to hSLC protocols cultured 3D cell aggregates in a mini-spin bioreactor during the IM and hSLC differentiation stages, resulting in the formation of steroidogenic, mesonephric, and even vascular cells within the hSLC organoids (Pryzhkova *et al.*, 2022). This enhanced level of cellular 3D organization, possibly driven by bioreactor-induced physical stimulation, highlights the importance of using 3D culture methods and mechanical cues during future protocol development.

During directed growth factor hGLC differentiation, Hart *et al.* (2022) and Lan *et al.* (2013) applied a similar approach by aggregating hPSCs into embryoid bodies, followed by induction of a gastrulation-like or IM-like fate, and subsequent attachment culture during differentiation (Table 2, ‘Directed growth factor’). Alternatively, Liu *et al.* (2016) performed monolayer culture with sequential growth factor addition (Table 2, ‘Directed growth factor’). Apart from factors discussed in the (intermediate) mesoderm differentiation section, a striking similarity between these three protocols was the use of GFs primarily associated with post-natal granulosa cell development, like Estradiol (E2), AMH, Inhibin-alpha/beta, TGF-alpha/beta, EGF, RA, HGH, FSH BMP15, GDF9, BMP4, FGF2, and FST (Fig. 3, ‘Directed’, hGLC, Supplementary Table S1). E2, AMH, FSH, Inhibin-alpha/beta, and TGF-beta were used in two of the three directed hGLC protocols, although with varying timing and duration of administration (Fig. 3, ‘Directed’, Table 2 ‘Directed growth factor’). Liu *et al.* (2016) used different combinations of these factors as well as RA and HGH, observing expression of AMH, FSHR, and Inhibin-alpha/beta, markers characteristic for post-natal granulosa cells (Vannier *et al.*, 1996; Sidis *et al.*, 1998; Anttonen *et al.*, 2011; Yding Andersen, 2017). Hart *et al.* (2022) developed two separate protocols combining E2, AMH, and Inhibin alpha/beta with either TGF-alpha/beta or BMP15 and GDF9 during the mesoderm-like cell to hGLC differentiation step (Table 2 ‘Directed growth factor’, Fig. 3), revealing induction of mural granulosa cell marker genes based on scRNA-seq data. Alternatively, Lan *et al.* (2013), used FGF2, BMP4, and FST for 6 days during their final differentiation step, inducing *FOXL2*, *AMHR2*, and FSHR expression (Table 2, ‘Directed growth factor’). Both Hart *et al.* (2022) and Lan *et al.* (2013) measured AMH and E2 secretion from the hGLC, confirming a post-natal phenotype. Remarkably, the hGLCs generated by Liu *et al.* (2016) were transplanted into a PCOS mouse model, resulting in a reduced atretic follicle number and providing a rare example of functional assessment of the differentiated niche. The lack of comparable analyses in other directed growth factor hGLC differentiation studies, combined with differences between performed analyses and growth factor combinations makes clear recommendations on promising GFs difficult.

The effect of utilizing growth factors with post-natal roles while mimicking embryonic differentiation stages *in vitro* is unclear, but might be critical for supporting late hGLC differentiation steps. This speculation aligns with the mural granulosa cell-like expression pattern observed by Hart *et al.* (2022). BMP15 is primarily associated with inducing a cumulus cell phenotype during post-natal follicle development as described in the *in vivo* section above. However, BMP15 can evidently promote a mural phenotype *in vitro*. Additionally, some factors included in the directed growth factor hGLC differentiation protocols are known to inhibit each other *in vivo*, such as AMH versus FSH or FST versus BMP4/15 (Fig. 3). This strategy may reflect an effort to replicate the fine-tuned regulation observed *in vivo*, where complex interplay between opposing signals supports cellular differentiation.

### Sertoli- or granulosa-like cell fate induction through directed transcription factor overexpression

An alternative method for directed hSLCs or hGLCs differentiation is by transcription factor overexpression, described in three protocols (Fig. 4, Tables 1 and 2, ‘Directed overexpression’) (Jung *et al.*, 2017; Liang *et al.*, 2019; Pierson Smela *et al.*, 2023). Liang *et al.* (2019) demonstrated that overexpressing *GATA4* and *NR5A1* in hESC-derived fibroblasts resulted in hSLCs positive for AMH and SOX9, as well as mature Sertoli cell markers like *CDKN1B* and *CLU* (Table 2, ‘Directed overexpression’). Unlike hESCs, these hSLCs were able to support mouse spermatogonia in culture, although only for 48 h. Similarly, Pierson Smela *et al.* (2023) screened for hGLC fate-inducing transcription factors in hiPSCs (Fig. 4). They found that overexpression of *NR5A1*, *RUNX1*, *GATA4*, and *FOXL2* in hiPSCs generated hGLCs that produced E2, but not Progesterone (P4) in response to FSH stimulation (Table 2, ‘Directed overexpression’). These hGLCs were co-cultured with hPGCLCs, leading to more rapid hPGCLCs maturation, indicated by DAZL expression, compared to hPGCLCs co-cultured with primary mouse foetal somatic cells. Despite a significant decline of germ cells over time, they observed antral-stage follicles with FOXL2 and AMHR2 positive hGLCs by Day 70 of culture. In a follow-up study, hGLCs were generated by overexpressing *NR5A1*, *GATA4*, and *RUNX2* (more mature) or *NR5A1*, *GATA4*, RUNX1, and *FOXL2* (foetal-like) (Piechota *et al.*, 2023). The mature hGLCs achieved higher oocyte maturation rates than the foetal-like hGLCs and significantly outperformed commercial *in vitro* maturation media in promoting metaphase II oocyte formation, demonstrating their functionality.

As an alternative to overexpressing somatic fate regulators, Jung *et al.* (2017) reported that overexpressing germ cell markers *DAZL* and *BOULE* in hiPSCs, followed by culturing in the presence of GDF9 and BMP15, also led to the formation of a subfraction of hGLCs (Fig. 4, Table 2, ‘Directed overexpression’). These hGLCs expressed *RSPO1*, *CYP19A*, and AMH. Notably, upon transplantation of the generated follicle-like structures (containing the hGLCs and oocyte-like cells) under the kidney capsule of recipient mice, follicular morphology, and AMH expression were retained, and E2 secretion was observed. This is remarkable, given the known distinct origins of germ cells and somatic lineages, and therefore, conceptually, not the most promising approach for efficient hGLC induction.

Overexpression of somatic lineage-inducing transcription factors is therefore more favourable, as hSLCs and hGLCs generated through this approach were able to promote hPGCLC maturation. Notably, *GATA4* and *NR5A1* overexpression was applied to generate both hSLCs and hGLCs (Fig. 4), demonstrating that these bipotential gonad markers are crucial for differentiating both lineages *in vitro*. Additionally, this approach offers the potential for faster hSLC or hGLC fate induction, as evidenced by shorter duration of the directed overexpression protocols (Tables 1 and 2). However, great care should be taken to ensure that no artifacts are introduced by assessing both reproducibility and the transcriptomic status of the differentiated cells by scRNA-seq, as performed by Pierson Smela *et al.* (2023). If these requirements are met, this approach offers the potential for therapeutic applications, but is less suitable for addressing developmental questions due to its bypassing of *in vivo* pathways.

## Discussion

Our assessment of current hPSCs to hSLCs and hGLCs differentiation protocols in the context of their *in vivo* development, has demonstrated the possibility to induce multiple Sertoli or granulosa cell marker genes by sequential or directed growth factor-based approaches, or through directed overexpression of key transcription factors. However, several challenges remain in the characterization, functional validation and advancement of these models, as discussed below.

### Challenges in advancing human *in vitro* gonadal models

#### In vivo knowledge gaps leading to obstacles in protocol optimization and reproducibility

All discussed hPSCs to hSLC or hGLC differentiation protocols are impaired by knowledge gaps in human gonadal development. Current mechanistic understandings, largely derived from murine studies, may overlook crucial species-specific differences (Garcia-Alonso *et al.*, 2022). Moreover, fundamental aspects of gonadal development remain unclear, with ongoing debate over whether gonadal progenitors in mice originate from the IM or the splanchnic LPM, while the identity of human progenitors is even less defined (Sasaki *et al.*, 2021; Neirijnck *et al.*, 2023). The unknown positioning of these progenitors along the embryonic axes and the associated exposure to signalling factors complicate *in vitro* modelling attempts, necessitating deeper exploration of the molecular events during human gonadal development. This knowledge gap complicates the prediction of potential concentration-dependent effects or interactions between these factors, leading to protocol optimization through trial and error. This approach is not only labour-intensive and costly, but also confounded by stochastic effects and variability in unknown factors leading to poor reproducibility. As a result, the advancement towards therapeutic applications which require precision is currently limited.

#### Limited characterization of differentiated cells

So far, two directed overexpression studies have compared differentiated hSLCs or hGLCs to their *in vivo* counterparts through a transcriptome-wide analysis (Liang *et al.*, 2019; Pierson Smela *et al.*, 2023). The lack of similar analyses in other studies limits the ability to verify the identity of *in vitro*-derived cell types and hinders the identification of optimal progenitors for generating hSLCs and hGLCs *in vitro*. Functional evaluation of the intermediate differentiated states remains technically challenging due to limited knowledge of these steps. However, hSLCs or hGLCs induction provides evidence of previous successful differentiation towards gastrulation-, IM-, and bipotential gonad-like stages. Functional evaluation of hSLCs or hGLCs was limited to four studies, assessing their ability to support hPGCLC maturation, sustain mouse spermatogonial viability, reduce atretic follicles in a PCOS mouse model, or form follicles under mouse kidney capsules (Liu *et al.*, 2016; Jung *et al.*, 2017; Liang *et al.*, 2019; Pierson Smela *et al.*, 2023). Nonetheless, the ability to support hPGCLC maturation up to complete meiosis or further remains the most critical functional evaluation, and this functionality has not been shown for either hSLCs or hGLCs.

### Further considerations and possibilities for improvement

#### Emerging innovations for advancing gonadal development research

Multi-omics datasets provide a powerful foundation for subsequent research. Human embryonic and genital ridge scRNA-seq data contribute to investigating starting points (Li *et al.*, 2017a; Tyser *et al.*, 2021; Garcia-Alonso *et al.*, 2022; Lardenois *et al.*, 2023; Zeng *et al.*, 2023). *In silico* tools for trajectory inference and cell–cell communication prediction from these data can offer insights into cellular differentiation dynamics in early gonadal development (Saelens *et al.*, 2019; Dimitrov *et al.*, 2022). Existing scRNA-seq datasets of human gonadal development could be analysed with these tools, as performed for cell–cell signalling involved in germ cell differentiation (Garcia-Alonso *et al.*, 2022). These approaches can be complemented by proteomics, DNA methylation, and assay for transposase-accessible chromatin sequencing (Saelens *et al.*, 2019; Dimitrov *et al.*, 2022). Additionally, spatial transcriptomics have proven valuable for characterizing human gastrulation and foetal ovarian differentiation, and should be performed for other stages of gonadal development (Garcia-Alonso *et al.*, 2022; Xiao *et al.*, 2024; Cui *et al.*, 2025).

Despite the availability of advanced ‘omics’ approaches, experimental hypothesis validation remains crucial and currently largely depends on animal models. Recent advancements, such as barcode-based tracing, DCM-time machine technology, and DamID, enable efficient tracking of developmental cell trajectories in mouse models (van Steensel and Henikoff, 2000; Boers *et al.*, 2023; Li *et al.*, 2024). These approaches offer the potential for a faster and more accurate understanding of gonadal somatic cell differentiation in mice and can aid the development of robust human *in vitro* models.

#### Pluripotent state transitions: from naïve to primed

Selecting the appropriate starting material for *in vitro* differentiation is crucial, considering factors such as the stem cell pluripotent state. PSCs can reside in a naïve state, corresponding to the pre-implantation epiblast, or a primed state, corresponding to the post-implantation epiblast (Nichols and Smith, 2009). Both hESCs and hiPSCs are considered to be in a primed state (Nichols and Smith, 2009; Kilens *et al.*, 2018). In mice, the transition from naïve to primed pluripotency has been shown to be crucial for differentiation into both germ and somatic lineages, and similar evidence is emerging for hPSCs *in vitro* (Hayashi *et al.*, 2011; Kalkan *et al.*, 2017; Rostovskaya *et al.*, 2019; Yoshino *et al.*, 2021; Alves-Lopes *et al.*, 2023). Additionally, hESCs and hiPSCs exhibit differences in their epigenetic profiles, influenced by persistent DNA methylation patterns retained from the somatic lineage from which hiPSCs are derived (Kim *et al.*, 2010; Lister *et al.*, 2011). hPSCs can be converted to naïve pluripotency, resulting in lower overall DNA methylation levels, and potentially overcoming epigenetic barriers hindering hiPSCs differentiation (Hanna *et al.*, 2010; Chan *et al.*, 2013; Gafni *et al.*, 2013; Takashima *et al.*, 2014; Theunissen *et al.*, 2014; Ware *et al.*, 2014). Currently, the influence of pluripotent state on gonadal somatic niche differentiation remains unclear, warranting studies that examine pluripotency states for their differentiation potential, as performed for hPGCLCs (Alves-Lopes *et al.*, 2023).

#### Appropriate 3D structure and cellular context

Beyond intracellular and intercellular factors, external physical and mechanic cues play a critical role in regulating differentiation. Only one of the current hSLC differentiation protocols used advanced 3D culture in a mini-spin bioreactor, achieving higher-order morphogenesis and vascularization (Pryzhkova *et al.*, 2022). This improvement of spatial organization through 3D culture may better mimic the *in vivo* tissue microenvironment (Jensen and Teng, 2020; Cacciamali *et al.*, 2022). Insights for enhancing 3D culture methods during final stages of *in vitro* hPSC differentiation can be gained from *ex vivo* culture protocols for human primary foetal material, which include systems like floating, air–liquid interface, hanging drop, and matrix-based gradient culture (Le Bouffant *et al.*, 2010; Jørgensen *et al.*, 2015; Frydman *et al.*, 2017; Macdonald *et al.*, 2018; Oliver *et al.*, 2021; Mizuta *et al.*, 2022). Additionally, mechanical cues in culture systems can be modulated using materials such as soft agar, hyaluronan-based hydrogels, synthetic polymers, and decellularized gonadal extracellular matrix, or even microfluidic organ-on-a-chip technology (Stukenborg *et al.*, 2008; Lee *et al.*, 2011; Shikanov *et al.*, 2011; Baert *et al.*, 2015; Vermeulen *et al.*, 2018). These technical adaptations may enhance maturation and overall functionality of human gonadal niche differentiation systems (Low *et al.*, 2021; Corsini and Knoblich, 2022; Lee *et al.*, 2023).

#### Recommendations for protocol development

To advance *in vitro* gonadal niche models, future protocols should integrate several key considerations. Prior to differentiation, hPSC maintenance requires strict culture conditions to preserve pluripotency and genomic stability. While ISSCR guidelines provide standards for ethical sourcing, documentation, and validation, the protocols discussed in this review do not address these aspects (International Society for Stem Cell Research, 2021). Additional greater standardization in culture conditions, including substrate selection, media composition, and quality control measures, is therefore necessary (Geraghty *et al.*, 2014; Lovell-Badge *et al.*, 2021).

During differentiation, multiple XX and XY cell lines should be examined to account for sex- and cell line-specific variability. We suggest the use of omics datasets to guide a more systematic selection and testing of new growth factors, using available tools for trajectory inference and cell–cell signalling analyses. While we have proposed various growth factors and combinations thereof for different stages of *in vitro* differentiation, it is crucial to optimize these for each step and cell line. This optimization should involve evaluating a panel of marker genes for both desired and undesired lineages.

The use of reporter cell lines or specific cell surface markers can yield homogenous cell populations. However, it is important to assess whether supportive cell types (e.g. Leydig, theca, or peritubular cells) are present in the negative fraction, as these may enhance niche formation for IVG. Critically, the differentiated hSLCs or hGLCs must be evaluated for their capacity to support hPGCLC maturation, ensuring developmental stage alignment between the niche and hPGCLCs. Ultimately, researchers should carefully align their study design with specific research goals, selecting appropriate validation assays.

### Co-culture systems of hSLC or hGLC with hPGCLCs and IVG

Combining hSLCs or hGLCs with hPGCLCs in co-culture systems provides a robust *in vitro* gonadal development model. While mESC-derived granulosa cells have successfully supported mPGCLCs leading to fertilization (Yoshino *et al.*, 2021), similar progress with PSC-derived Sertoli cells remains elusive, even in rodents. The current lack of a functional hPSC-derived gonadal niche necessitates reliance on mouse somatic niches, generating xenogeneic reconstituted (xr) testes or xr-ovaries that achieve partial hPGCLC maturation into oogonia or prospermatogonia (Yamashiro *et al.*, 2018; Hwang *et al.*, 2020; Kobayashi *et al.*, 2022). Recent advances, including hPGCLC co-culture with primary foetal ovarian cells or hindgut organoids (Alves-Lopes *et al.*, 2023; Overeem *et al.*, 2023), demonstrate maturation levels comparable to transcription factor-induced hGLC co-cultures (Pierson Smela *et al.*, 2023). However, neither strategy has overcome the critical barrier of meiotic entry. The pre-migratory state of most hPGCLCs poses a key challenge, as optimal crosstalk with hSLCs or hGLCs requires synchronization of their developmental stages.

#### From sex determination to IVG

Ideally, a unified protocol would fully recapitulate sex determination in both XY and XX systems, generating gonadal somatic progenitors that diverge into hSLCs or hGLCs in a cell-autonomous manner. Achieving successful IVG with hSLCs or hGLCs requires overcoming additional barriers related to pubertal Sertoli cell maturation and different granulosa cell subtypes, respectively, in the appropriate context with germ cells that properly progress through gametogenesis. Finally, for both XY and XX IVG, the inclusion of additional gonadal somatic lineages beyond hSLCs and hGLCs might be necessary to ensure efficient gonadal morphogenesis and complete germ cell maturation.

### Future implications and potential applications

The successful development of *in vitro* models for human gonadal development would impact both fundamental research and clinical implications. These models could provide insights into human sex determination and gonadal development, offering a platform to study underlying mechanisms of DSD and infertility, paving the way for new treatments or interventions. Notably, the use of hiPSCs instead of hESCs presents potential for application of patient-specific IVG in fertility treatments. However, the ethical and safety considerations of this approach may prevent its practical application (Notini *et al.*, 2020; Adashi *et al.*, 2024). Further research and ethical discussion are needed to explore the full potential of IVG in reproductive medicine. Ultimately, *in vitro* hPSC-based gonadal differentiation models and IVG would enhance our understanding of human biology and open new avenues for diagnosing, understanding and, potentially, treating various reproductive and developmental disorders.

## Supplementary Material

dmaf012_Supplementary_Data

## Data Availability

No new data was analysed or generated during this study.
